# Targeted genetic screening in mice through haploid embryonic stem cells identifies critical genes in bone development

**DOI:** 10.1371/journal.pbio.3000350

**Published:** 2019-07-02

**Authors:** Meizhu Bai, Yujiao Han, Yuxuan Wu, Jiaoyang Liao, Lin Li, Lijun Wang, Qing Li, Wenhui Xing, Luonan Chen, Weiguo Zou, Jinsong Li

**Affiliations:** 1 State Key Laboratory of Cell Biology, Shanghai Key Laboratory of Molecular Andrology, CAS Center for Excellence in Molecular Cell Science, Shanghai Institute of Biochemistry and Cell Biology, Chinese Academy of Sciences, University of Chinese Academy of Sciences, Shanghai, China; 2 State Key Laboratory of Cell Biology, CAS Center for Excellence in Molecular Cell Science, Shanghai Institute of Biochemistry and Cell Biology, Chinese Academy of Sciences, University of Chinese Academy of Sciences, Shanghai, China; 3 Shanghai Key Laboratory of Regulatory Biology, Institute of Biomedical Sciences and School of Life Sciences, East China Normal University, Shanghai, China; 4 Key Laboratory of Systems Biology, CAS Center for Excellence in Molecular Cell Science, Innovation Center for Cell Signaling Network, Institute of Biochemistry and Cell Biology, Shanghai Institutes for Biological Sciences, Chinese Academy of Sciences, Shanghai, China; 5 School of Life Science and Technology, Shanghai Tech University, Shanghai, China; Universitat Wien, AUSTRIA

## Abstract

Mutagenic screening is powerful for identifying key genes involved in developmental processes. However, such screens are successful only in lower organisms. Here, we develop a targeted genetic screening approach in mice through combining androgenetic haploid embryonic stem cells (AG-haESCs) and clustered regularly interspaced palindromic repeats/CRISPR-associated protein 9 (CRISPR-Cas9) technology. We produced a mutant semi-cloned (SC) mice pool by oocyte injection of AG-haESCs carrying constitutively expressed Cas9 and an single guide RNA (sgRNA) library targeting 72 preselected genes in one step and screened for bone-development–related genes through skeletal analysis at birth. This yielded 4 genes: *Zic1* and *Clec11a*, which are required for bone development, and *Rln1* and *Irx5*, which had not been previously considered. Whereas *Rln1*^*−/−*^ mice exhibited small skeletal size only at birth, *Irx5*^*−/−*^ mice showed skeletal abnormalities both in postnatal and adult phases due to decreased bone mass and increased bone marrow adipogenesis. Mechanistically, iroquois homeobox 5 (IRX5) promotes osteoblastogenesis and inhibits adipogenesis by suppressing peroxisome proliferator activated receptor γ (PPARγ) activation. Thus, AG-haESC-mediated functional mutagenic screening opens new avenues for genetic interrogation of developmental processes in mice.

## Abstract 2

Androgenetic haploid embryonic stem cells expressing Cas9 and a medium-scale sgRNA library provide a means to mutate genes at the organism level, enabling knockout-based screening in mammalian systems. Applying this approach successfully identified Irx5 as a regulator of bone development.

## Introduction

Large-scale recessive genetic screening is a powerful approach used to identify genes underlying biological processes. This strategy has been successful in lower organisms such as yeast (*Saccharomyces cerevisiae*) [1–3] and nematode (*Caenorhabditis elegans*) [4]. In mammalian organisms, such functional screening is challenging due to their diploid genomes [5]. RNA interference (RNAi) is currently used for genome-scale loss-of-function screening in mammalian cells by targeting mRNA [6, 7]; however, this approach is often insufficient to suppress the gene expression and has off-target effects on other mRNAs [8]. The clustered regularly interspaced palindromic repeats/CRISPR-associated protein 9 (CRISPR-Cas9) system from bacteria has been applied to efficient loss-of-function screening in mouse and human cells [9–11]. Recently, this system was used to assay genes in tumor growth and metastasis [12]. Nevertheless, CRISPR-Cas9–mediated genome-scale screen has been so far employed only at the cellular level, limiting its application to cell-based phenotypes.

The generation of mammalian haploid embryonic stem cells (haESCs) from parthenogenetic or androgenetic blastocysts [13–16] provides an ideal tool for genetic analysis at the cellular level [17–20]. Androgenetic haESCs (AG-haESCs), whose genome is from the sperm [21–23], support full-term development of embryos upon injection into mature oocytes (intracytoplasmic AG-haESCs injection [ICAHCI]), resulting in live animals referred to as semi-cloned (SC) animals. Importantly, double knockout (DKO) AG-haESCs of *H19-DMR* and *IG-DMR* (DKO-AG-haESCs) exhibited “fertilization” capacity comparable to round spermatids, reaching a success rate of 20% of transferred SC embryos [24]. Moreover, DKO-AG-haESCs carrying constitutively expressed Cas9 and single guide RNA (sgRNA) library allow one-step generation of mutant mice, showing the potential for functional mutagenic screening at the organism level in mice [24, 25].

Mammalian skeletal development and remodeling are critical for the maintenance of the biomechanical properties of bone [26]. Osteoblasts, originated from mesenchymal stem cells, are responsible for the formation and mineralization of the skeleton. Osteoclasts, the multinucleated giant cells from myeloid lineage can resorb the existing bone through an acidic and enzymatic process [27]. Imbalance between bone resorption and bone formation during bone development and remodeling will lead to the pathogenesis of skeletal disorders, such as osteoporosis and osteopetrosis [26]. Although many genes have been described to regulate osteoblast and osteoclast function, numerous complex regulatory networks that control bone development and remodeling remain unidentified. Generally, genes critical for bone development and remodeling are identified using genetic mouse models carrying a single mutant gene [28]. However, this approach is limited by the rate at which new genes are identified. Meanwhile, some well-studied genes may have unknown functions in bone development. To systematically identify the critical genes associated with osteogenesis, a high-throughput forward genetic screen has been established in zebrafish via N-ethyl-N-nitrosourea (ENU)-induced random mutation [29]. In mammals, high-throughput small interfering RNA (siRNA) library screens to identify osteogenic factors could be carried out only in cultured cells [30, 31]. Therefore, establishment of a high-throughput targeted genetic screening approach in mice will accelerate the identification of novel genes and signaling pathways or novel functions of well-known genes in bone development [30].

In this study, we established an in vivo genetic screening strategy to identify genes involved in bone development based on the combined application of ICAHCI technology and the CRISPR-Cas9 library. We first improved the efficiency of biallelic mutant mice generation by deriving a haploid cell line carrying constitutively expressed Cas9 with high DNA cleavage ability. We further demonstrated that these cells carrying an sgRNA library targeting 72 preselected genes that may be involved in bone development could be used to generate mice with different mutations. Through the skeletal analysis at birth, we identified 4 genes that appear to affect skeletal development, one of which is *Irx5*. We showed that *Irx5* acts as a positive regulator of bone development by suppressing peroxisome proliferator activated receptor γ (PPARγ), leading to enhanced osteoblast differentiation and reduced adipocyte differentiation during bone development.

## Results

### haESCs carrying constitutively expressed Cas9 and sgRNA efficiently generate mutant mice

**Fig 1:**
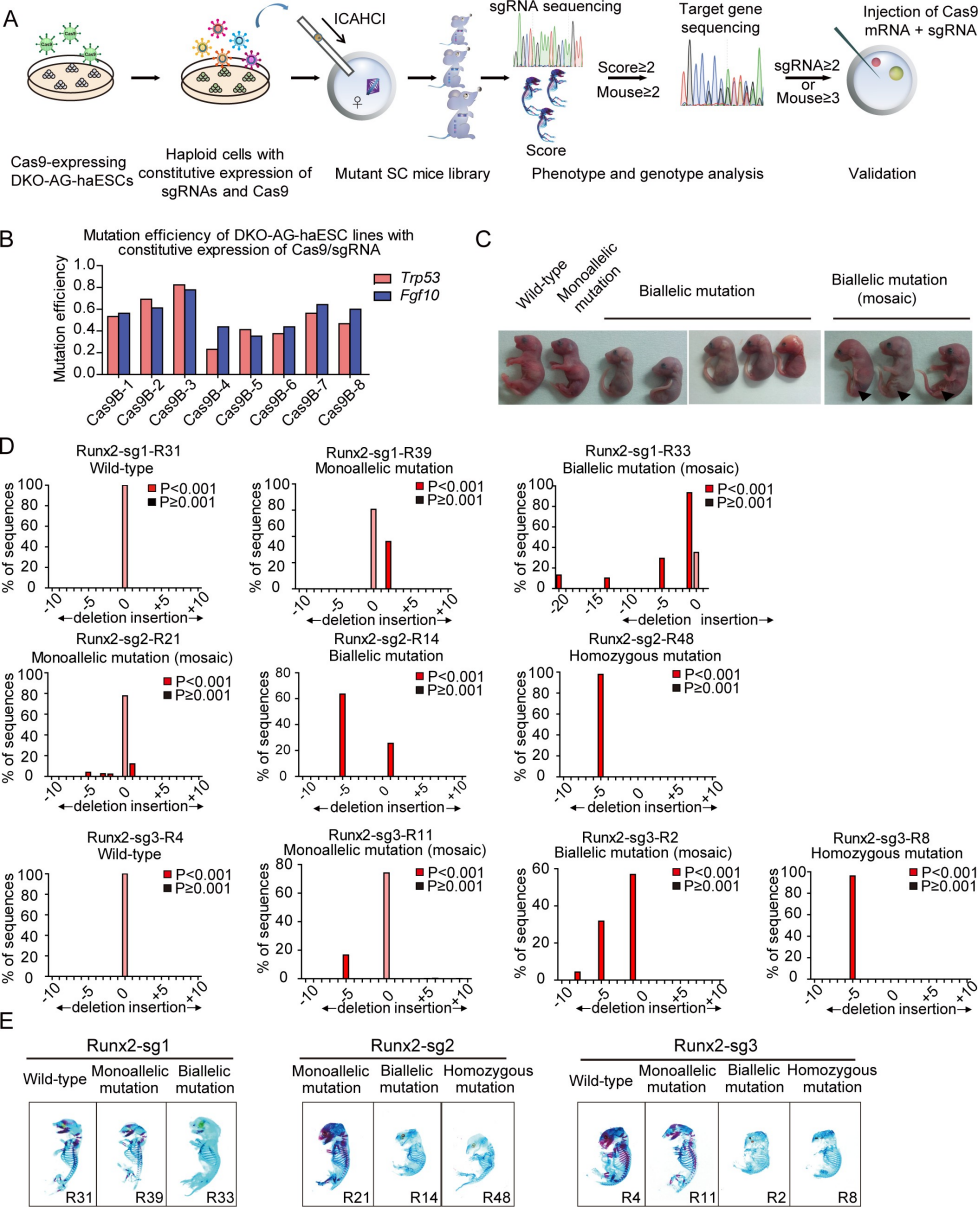
DKO-AG-haESCs carrying Cas9 and an sgRNA support efficient generation of biallelic mutant SC mice. (A) Schematic of genetic screening in mice via ICAHCI using DKO-AG-haESCs carrying constitutively expressed Cas9 and sgRNAs. (B) Mutation efficiency of DKO-AG-haESC lines carrying constitutively expressed Cas9 and an sgRNA (*Trp53* or *Fgf10*) expanded from single-cell clones (Cas9B-1–8). Data associated with this figure can be found in S1 Data. (C) SC pups carrying constitutively expressed Cas9 and an sgRNA-targeting *Fgf10* gene generated by ICAHCI. Arrows indicate the limb defects in mosaic pups with *Fgf10* gene biallelic mutation. (D) TIDE mediated sequence analysis of SC pups carrying constitutively expressed Cas9 and sgRNA targeting *Runx2*. Sanger sequencing is shown in S1G Fig, and the relative phenotype of these mice is shown in Fig 1E and S1H Fig. Data associated with this figure can be found in S1 Data and S1 Table. (E) Whole-mount staining of SC pups carrying constitutively expressed Cas9 and sgRNA targeting *Runx2* using alcian blue and alizarin red. The relative genotype analysis is shown in Fig 1D and S1 Table. Cas9, CRISPR-associated protein 9; DKO-AG-haESC, double knockout androgenetic haploid embryonic stem cell; ICAHCI, intracytoplasmic AG-haESCs injection; Runx2, runt related transcription factor 2; SC, semi-cloned; sgRNA, single guide RNA; TIDE, tracking of indels by decomposition.

Functional mutagenic screening is a powerful tool for developmental studies; however, this is still an unmet need in mammals. Recently, we demonstrated the use of DKO-AG-haESCs carrying constitutively expressed Cas9 and an sgRNA library to generate biallelic mutant mice through ICAHCI [24], showing the possibility of applying this strategy in a defined developmental context to effectively identify critical genes from a targeted library. In this study, we propose a workflow to screen for important genes involved in bone development, including construction of 1 medium-scale sgRNA library, derivation of a haploid cell line carrying the library, production of SC pups via ICAHCI, analysis of skeletal phenotype by whole-mount staining, determination of bone-development–related genes, validation of the candidate gene by establishing mouse lines, and illumination of the underlying mechanisms (Fig 1A).

Our previous study demonstrated the use of DKO-AG-haESCs carrying constitutively expressed Cas9 and an sgRNA library to generate biallelic mutant mice through ICAHCI [24]. However, the overall mutant efficiency in mice was around 30% of born SC pups (83/272) [24]. To further increase the biallelic mutation rate of SC pups, we first attempted to derive haploid cells with high Cas9 activity. After transfection of DKO-AG-haESCs from the *IG*^*ΔDMR*^-*H19*^*ΔDMR*^*-* AGH-2 cell line [24] with lentiviral Cas9, a total of 8 cell lines (termed Cas9B-1 to Cas9B-8, respectively) carrying constitutively expressed Cas9 were generated by single-cell expansion. To test their cleavage efficiency, the DKO-AG-haESCs were transfected with lentivirus harboring sgRNA targeting *Trp53* or a well-known developmental gene, *Fgf10* [32] (S1A and S1B Fig). We found that the Cas9B-3 cells showed the highest Cas9 cleavage efficiency in haploid cells (Fig 1B and S1C Fig). To ensure the in vivo cleavage of haploid cells carrying constitutively expressed Cas9, we next injected haESCs from Cas9B-3 harboring the sgRNA targeting *Fgf10* into oocytes (S1D Fig). ICAHCI led to the birth of 10 SC pups (Fig 1C and Table 1), of which 8 died at birth. Interestingly, Sanger sequencing of PCR products amplified from sgRNA-targeted sites using tail genomic DNA indicated that all of the 8 dead mice (an efficiency of 80% of born SC pups) carried mutant *Fgf10* (S1E Fig). Consistently, all of them exhibited limb defects (Fig 1C), the phenotype observed in *Fgf10*^−/−^ mice [32, 33]. Subsequently, the Cas9B-3 cell line was used for the targeting experiments.

**Table 1:** Summary of SC mice derived from DKO-AG-haESCs carrying constitutively expressed Cas9 and sgRNAs targeting *Fgf10*, *Runx2*, or genes in BD library.

sgRNA		No. of embryos transferred	No. of SC pups (% of transferred embryos)	No. of SC pups carrying 1 sgRNA	No. of SC pups without sgRNA	No. of SC pups carrying sgRNA (*n* ≥ 2)	No. of SC pups with homozygous mutation (% of SC pups carrying 1 sgRNA)	No. of SC pups with biallelic mutation (% of SC pups carrying 1 sgRNA)	No. of SC pups with monoallelic mutation (% of SC pups carrying 1 sgRNA)	No. of WT SC pups (% of SC pups carrying 1 sgRNA)
*Fgf10*		73	10 (13.7%)	10	ND^a^	ND	0	8 (80.0%)	1 (10.0%)	1 (10.0%)
*Runx2*	Sg-1	107	15 (14.0%)	15	ND	ND	0	3 (20.0%)	5 (33.3%)	7 (46.7%)
	Sg-2	158	23 (14.6%)	23	ND	ND	4 (17.4%)	18 (78.3%)	1 (4.3%)	0
	Sg-3	72	13 (18.1%)	13	ND	ND	3 (23.1%)	7 (53.8%)	1 (7.7%)	2 (15.4%)
	Subtotal	337	51 (15.1%)	51	ND	ND	7 (13.7%)	28 (54.9%)	7 (13.7%)	9 (17.7%)
BD Library	Pretest	342	39 (11.4%)	38	1	0	3 (7.9%)	18 (47.4%)	8 (21.1%)	9 (23.6%)
	Total	3,601	426 (11.8%)	406	15	5	ND	ND	ND	ND

**Abbreviations:** BD, bone development related; Cas9, CRISPR-associated protein 9; DKO-AG-haESC, double knockout androgenetic haploid embryonic stem cell; ND, not determined; SC, semi-cloned; sgRNA, single guide RNA; WT, wild type

### Cas9B-3 cells carrying *Runx2* sgRNAs efficiently generate mutant mice with bone defects

We next investigated the feasibility of our system to study bone development by targeting the transcription factor *Runx2* that is essential for osteoblast differentiation and bone development [34–36]. Three sgRNAs were designed to target exon 3 or 4 of *Runx2* (termed Runx2-sg1, Runx2-sg2, and Runx2-sg3; S1F Fig) and individually transfected into Cas9B-3 cells to derive stable cell lines carrying each sgRNA for ICAHCI experiments. SC pups were generated at an average efficiency of 15.1% (Table 1), comparable to the efficiency reported in wild-type or transgenic DKO-AG-haESCs [24]. Genotyping analysis of tail DNA indicated that haploid cells with Runx2-sg2 and Runx2-sg3 efficiently produced mutant mice with homozygous or biallelic mutations (95.7% and 76.9% of born SC pups, respectively; Table 1). In contrast, Runx2-sg1 exhibited low cleavage efficiency in SC mice, indicating that sgRNA per se determines the mutation rate in mice [37]. A total of 51 SC pups were produced from 3 sgRNAs, of which 35 carried homozygous or biallelic mutations according to Sanger sequencing results (S1G Fig). Tracking of indels by decomposition (TIDE) [38] analysis indicated that more than 75% of sequences were mutated in mice with homozygous or biallelic mutations, whereas less than 50% of DNA sequences were mutated in mice with monoallelic mutation (Fig 1D and S1 Table). Meanwhile, TIDE analysis indicated that 16 out of 35 homozygous or biallelic mutant mice carried no in-frame mutations or/and wild-type sequences, suggesting that they were most likely loss-of-function mutant pups (Table 2 and S1 Table). The other SC pups carried a certain number of in-frame mutations or/and wild-type sequences, of which 9 were with in-frame mutations at a percentage less than 30% and 10 carried in-frame mutations or/and wild-type sequences at a percentage more than 30% (Table 2 and S1 Table). Furthermore, TIDE results showed that 15 SC pups with homozygous or biallelic mutations exhibited chimerism due to carrying more than 2 types of alleles (Table 2 and S1 Table), which is a common phenomenon observed in mutant mice generated by injection or electroporation of CRISPR-Cas9 into zygotes [39].

**Table 2:** Genotype analysis of homozygous or biallelic mutant SC mice carrying constitutively expressed Cas9 and sgRNA targeting *Runx2* or genes in BD library.

sgRNA	No. of biallelic or homozygous mutant SC mice without in-frame mutation and/or WT (%)		No. of biallelic or homozygous mutant SC mice with in-frame mutation and/or WT < 30% (%)		No. of biallelic or homozygous mutant SC mice with in-frame mutation and/or WT > 30% (%)	
	≤2 types of alleles	>2 types of alleles	≤2 types of alleles	>2 types of alleles	≤2 types of alleles	>2 types of alleles
Runx2-sg1	0/3 (0)	0/3 (0)	0/3 (0)	2/3 (66.7%)	0/3 (0)	1/3 (33.3%)
Runx2-sg2	11/22 (50.0)	0/22 (0)	0/22 (0)	3/22 (13.6)	4/22 (18.2)	4/22 (18.2)
Runx2-sg3	3/10 (30.0)	2/10 (20.0)	1/10 (10.0)	3/10 (30.0)	1/10 (10.0)	0/10 (0)
Average	14/35 (40.0)	2/35 (5.7)	1/35 (2.9)	8/35 (22.8)	5/35 (14.3)	5/35 (14.3)
Runx2-total	16/35 (45.7)		9/35 (25.7)		10/35 (28.6)	
BD pretest	3/21 (14.3)	1/21 (4.8)	3/21 (14.3)	6/21 (28.5)	3/21 (14.3)	5/21 (23.8)
BD pretest-total	4/21 (19.1)		9/21 (42.8)		8/21 (38.1)	

**Abbreviations:** BD, bone development related; Cas9, CRISPR-associated protein 9; SC, semi-cloned; sgRNA, single guide RNA; WT, wild type

We next performed the whole-mount skeletal staining of all 51 pups with alcian blue and alizarin red [30, 40] and graded them according to 5 bone-related phenotypes, including whole-mount size, calcification degree, and morphology of calvarial bone, vertebral bone, and long bone (Fig 1E; S1H Fig and S1 Table). The bone abnormality of each SC pup was scored based on the frequency of occurrence of the abnormality (for example, if 1 SC pup has 2 abnormities in the above 5 phenotypes, its abnormity score is 2). According to S1 Table, 33 of 35 homozygous or biallelic mutant mice were scored from 3 to 5 [34–36], whereas 6 of 7 monoallelic mutant mice were scored from 0 to 2, and all 9 wild-type mice were scored 0. Interestingly, 2 homozygous or biallelic mutant pups with over 65% in-frame mutant sequences were scored 1. Meanwhile, along with the increase of in-frame mutations in the SC pup, the bone abnormality score was inclined to decrease. In addition, we also observed that one monoallelic mutant pup was scored 3 although 60% of its sequences were wild type, probably caused by CRISPR-Cas9–mediated off-target effects or chimerism. Taken together, our data indicated high consistency between genotype and phenotype of resultant SC pups generated by our strategy, demonstrating that the combination of DKO-AG-haESCs and CRISPR-Cas9 can efficiently generate biallelic mutant SC pups via ICAHCI, and PCR-based genotyping results of tail genomic DNA can identify the mutations in the SC mice.

### Generation of DKO-AG-haESCs carrying an sgRNA library targeting a medium-scale collection of genes

**Fig 2:**
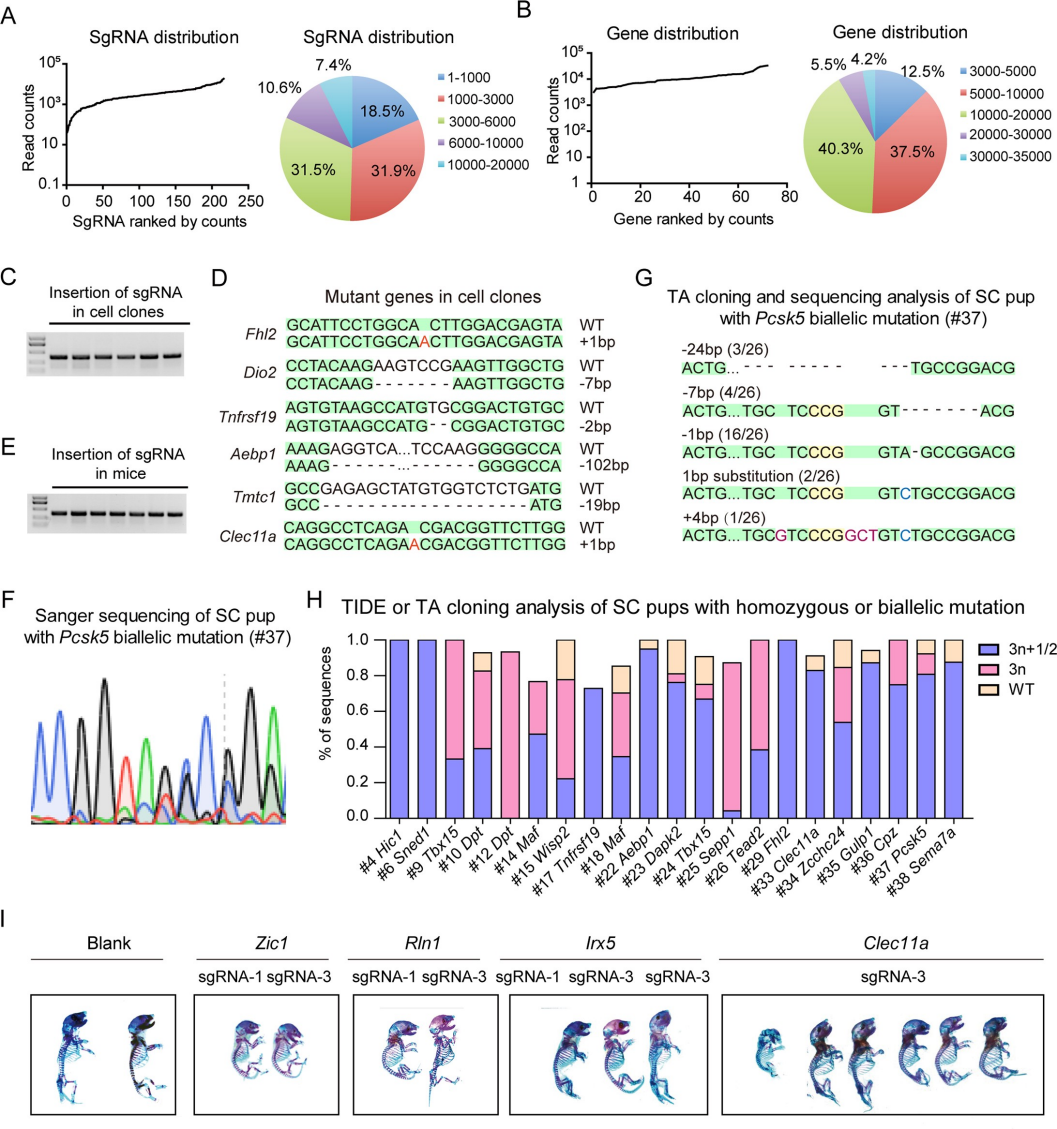
Cas9B-3-BD supports efficient generation of biallelic mutant SC mice in one step. (A–B) Deep sequencing analysis of Cas9B-3-BD cells carrying BD library based on the distribution of sgRNAs (A) or genes (B). Data associated with this figure can be found in S1 Data and S4 Table. (C) PCR analysis of sgRNA insertions in haploid cell clones expanded from single cells in Cas9B-3-BD. (D) Genotyping analysis of different cell clones picked up from Cas9B-3-BD. Deletions are indicated with (−); insertions are labeled in red; sequences shaded in green are the same as WT allele. (E) Identification of sgRNA insertions in SC mice by PCR analysis. (F) Sanger sequencing of SC pup (#37) with *Pcsk5* biallelic mutation generated by ICAHCI of Cas9B-3-BD cells. (G) TA cloning and sequencing analysis of the SC pup (#37) with *Pcsk5* gene mutation generated by ICAHCI of Cas9B-3-BD cells. Five types of alleles were detected from 1 PCR sample. Deletions are indicated with (−); insertions are labeled in red, and substitutions are labeled in blue; sequences shaded in green are the same as WT allele, and the PAM sites are shaded in yellow. (H) TIDE or TA cloning analysis of SC pups with homozygous or biallelic mutation generated by injection of Cas9B-3-BD cells into oocytes in the first 3 ICAHCI experiments. Data associated with this figure can be found in S1 Data and S7 Table. (I) Whole-mount staining of SC pups with sgRNA targeting *Zic1*, *Rln1*, *Irx5*, or *Clec11a* generated from Cas9B-3-BD cells at birth using alcian blue and alizarin red. AG-haESC, androgenetic haploid embryonic stem cell; BD, bone development related; bp, base pair; Cas9, CRISPR-associated protein 9; ICAHCI, intracytoplasmic AG-haESCs injection; PAM, protospacer adjacent motif; SC, semi-cloned; sgRNA, single guide RNA; TA, thymine and adenine; TIDE, tracking of indels by decomposition; WT, wild type.

After attaining high-efficiency production of biallelic SC pups from Cas9B-3 cells, we examined whether Cas9B-3 cells carrying a targeted sgRNA library could enable mutageneic screening in vivo through efficient production of biallelic SC mice in one step via ICAHCI. A total of 72 candidate genes that may play roles in bone development were selected for the screening based on the osteogenesis expression data sets in BioGPS gene portal system (S2 Table). The expression level of each gene relative to its median expression across all samples was calculated as shown in S2 Table. According to the analysis, out of 69 genes highly expressed in osteoblast, 23 were osteoblast specific. Meanwhile, the expression of 39 genes showed more than 2-fold increase or decrease during osteoblast differentiation (S2A Fig and S2 Table). We designed 3 sgRNAs for each candidate gene [41] and constructed 1 lentiviral sgRNA library containing a total of 216 sgRNAs (termed bone development related[BD] library; S3 Table). Fluorescence-activated cell sorting (FACS)-enriched haploid cells from the Cas9B-3 cell line were infected with the BD library according to an earlier protocol [24]. Briefly, transfected cells were selected by puromycin treatment 2 days after transfection, followed by expansion for several passages to derive a cell population termed Cas9B-3-BD. To determine the coverage of sgRNAs in the haploid cells carrying the library, we performed deep sequencing analysis and found that all of the 216 sgRNAs were present in the cells (Fig 2A and S4 Table). Although a small fraction of sgRNAs was under- or overrepresented, around 74% of sgRNAs fell within a 10-fold difference in frequency (Fig 2A). As for the genes, the percentage was up to 97% (Fig 2B and S4 Table). We further examined whether the genes were mutated by CRISPR-Cas9 in haploid cells by randomly analyzing 35 cell clones. Among them, 34 clones carried only 1 sgRNA, and DNA sequencing analysis indicated that the targeted genes were mutated in 27 clones, reflecting an efficient gene mutation by CRISPR-Cas9 in haploid cells (Fig 2C and 2D; S5 Table).

Based on the distribution of genes in the Cas9B-3-BD library determined by deep sequencing, we estimated the number of cells to cover a group of genes using multinomial distribution analysis. For a given number of genes and cells, we could estimate the probability under the condition that the number of cells would cover all genes. According to our data, the probability that 72 genes are covered with 700 cells is more than 70%, whereas the probability that 71 genes are covered with 500 cells is 71.7%. Moreover, the probability that 400 cells cover 69 genes is 86.9% (S6 Table). Above all, through biometric analysis based on the distribution of genes in a library, we provide a valuable reference to predict the number of mice required to cover a certain number of genes.

### ICAHCI-mediated targeted genetic screening in mice using Cas9B-3-BD cells

Next, we performed ICAHCI experiments using Cas9B-3-BD cells to generate mutant mice in 1 step according to the previously reported protocol [24]. We attempted to identify the critical genes involved in bone development through skeletal analysis of around 400 newborn SC pups that may cover 69 of the candidate genes with the probability of 86.9% based on S6 Table. To test the mutation rate in the resultant SC pups from Cas9B-3-BD cells, we analyzed a total of 39 SC pups generated from the first 3 ICAHCI experiments. The sgRNA insertion analysis indicated that 38 pups carried 1 sgRNA (Fig 2E and Table 1). Regular genotyping analysis by Sanger sequencing showed that 29 of them carried mutations at the sgRNA-targeted sites, in which 21 carried homozygous or biallelic mutations at an efficiency of 55.3% of born SC pups (Fig 2F; Table 1; S2B Fig and S7 Table), dramatically higher than the previously reported efficiency of 30.5% (83/272) [24]. TIDE or thymine and adenine (TA) cloning analysis showed that 13 out of 21 homozygous or biallelic mutant mice carried in-frame mutations/wild-type sequences of less than 30% of total sequences, and 8 were with a percentage of more than 30% (Fig 2G and 2H; Table 2 and S7 Table). Meanwhile, TIDE results showed that 12 SC pups carried more than 2 types of alleles (Fig 2H; Table 2 and S7 Table), consistent with our early observations when *Runx2* was targeted. Taken together, these results demonstrate that mutant mice can be efficiently generated via ICAHCI using Cas9B-3-BD cells that harbor a targeted sgRNA library and constitutively expressed Cas9.

A total of 426 SC pups (including 39 from the first 3 experiments) were obtained from Cas9B-3-BD cells in this study (Table 1 and S7 Table). Among them, 406 pups carried only 1 sgRNA covering all 72 genes, in which 28 genes were targeted by 1 sgRNA, 33 genes were targeted by 2 different sgRNAs, and 11 genes were targeted by all 3 sgRNAs (S7 Table). We performed whole-mount staining of all 426 SC pups at birth using alcian blue and alizarin red (S2C Fig) and characterized the bone phenotype of 406 SC pups with only 1 sgRNA (S7 Table). We checked 5 bone-related phenotypes, including whole-mount size, calcification degree, and morphology of calvarial bone, vertebral bone, and long bone. The bone abnormality of each SC pup was scored based on the frequency of occurrence of the abnormality, showing abnormal bone development in a total of 42 SC pups (score ≥ 2). Meanwhile, 13 of 15 (Blank) SC pups from Cas9B-3-BD cells that carried only Cas9 and the sgRNA backbone displayed no abnormalities, excluding the possibility that expressed Cas9 induced the born defects during development (S2C Fig and S7 Table). However, the other 2 exhibited abnormal bone phenotypes (13 with abnormal calcification and 90 with a small size and abnormal skulls), probably due to ICAHCI per se because a small ratio of SC embryos exhibited growth-retarded phenotypes [24]. Together with possibilities of off-target effects and chimerism induced by CRISPR-Cas9, we thought that the readout of in vivo genetic screening might be affected by many factors. Therefore, to enhance the accuracy of in vivo screening, we chose genes that were targeted by 2 different sgRNAs in at least 2 SC pups with score ≥ 2or by 1 sgRNA in at least 3 SC pups with score ≥ 2, for further analysis (S7 Table). This resulted in 4 genes: *Zic1*, *Clec11a*, *Rln1*, and *Irx5* in 13 SC pups (Fig 2I). As expected, DNA sequencing analysis confirmed the mutations in the sgRNA-targeted sites in these SC pups, and 10 out of 12 biallelic mutant pups carried in-frame mutations/wild-type sequences of less than 30% according to TIDE analysis (S7 Table).

Interestingly, *Zic1* is involved in bone development in that its deletion leads to skeletal abnormalities in the developing and adult mice [42]. Meanwhile, c-type lectin domain family 11 (CLEC11A)—a secreted sulfated glycoprotein and growth factor for hematopoietic stem cells [43–45], highly expressed in bone fragments [46]—has been recently demonstrated to be an osteogenic growth factor that promotes the maintenance of the adult skeleton [47]. Taken together, these data provide a proof of principle for the feasibility of in vivo genetic screen for bone development via the combination of ICAHCI and CRISPR-Cas9.

### Validation of *Rln1* and *Irx5* by the generation of mutant mouse lines

A previous study has shown that *Rln1* plays a functional role in osteoblastic differentiation in cultured cells [48], suggesting its potential role in bone development. *Irx5* was relatively highly expressed during osteoblast differentiation (S2 Table), and previous studies have shown that combined deletion of *Irx3* and *Irx5* leads to significant skeletal limb malformations [49] and mimics human iroquois homeobox 5 (IRX5) mutation-induced defective craniofacial development in mice [50, 51]. However, *Irx5* mutation alone leads to viable mice with normal fertility, although they are slightly smaller than their wild-type counterparts [52, 53], leaving an open question of whether *Irx5* is involved in bone development.

**Fig 3:**
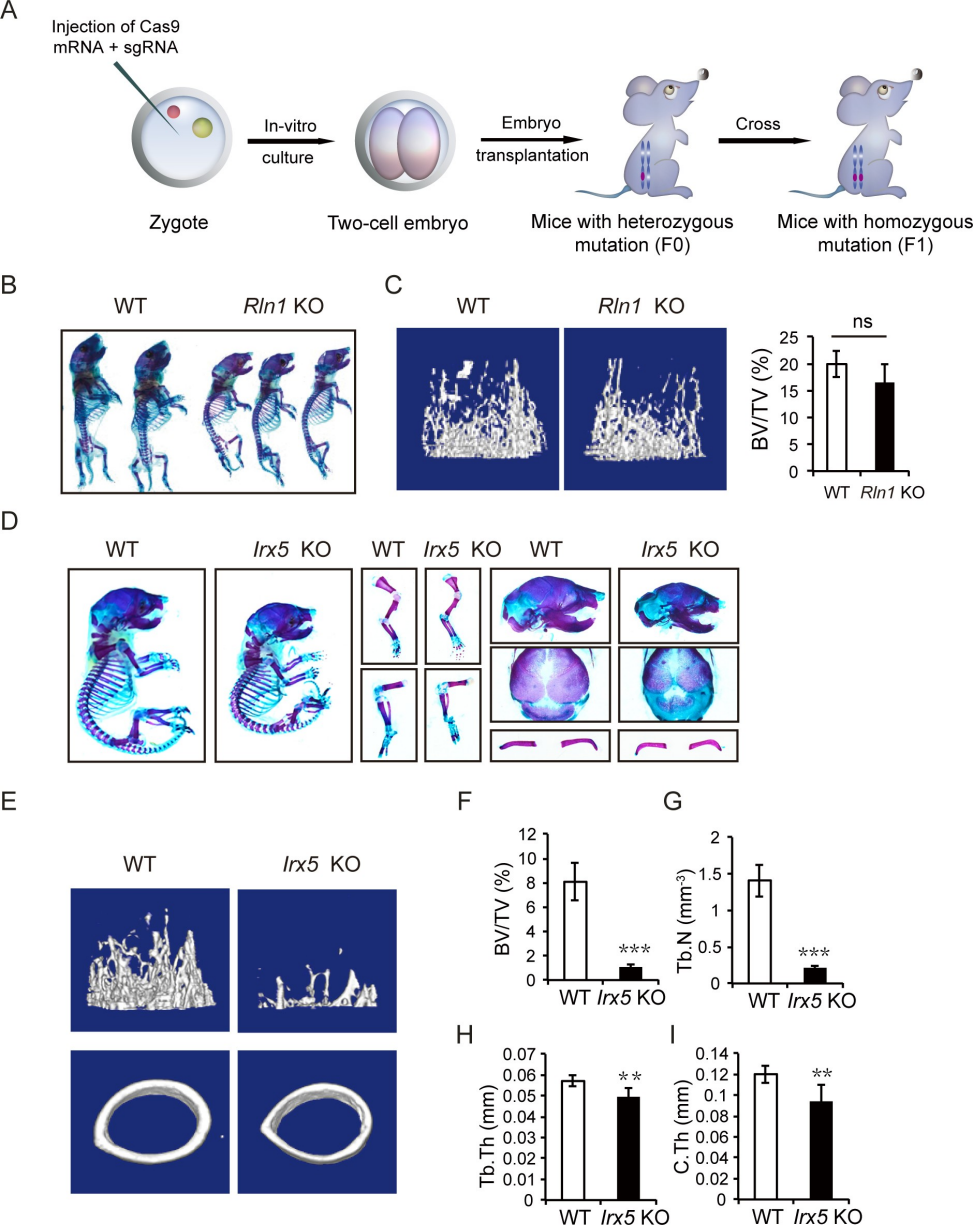
Validation of *Rln1* and *Irx5* by generation of mutant mouse lines. (A) Schemetic of *Rln1* and *Irx5* KO mouse line construction via zygote injection of Cas9 mRNA and sgRNA. (B) Whole-mount staining of WT and *Rln1* KO mice (F1) at P0 by alcian blue and alizarin red staining. (C) μCT analysis of distal femoral metaphysis from 12-week-old WT and *Rln1* KO mice. Representative three-dimensional reconstruction of μCT images (left) and the percentage of trabecular BV/TV (right). (D) Alcian blue and alizarin red staining of the whole skeletons, forelimb, hindlimb, calvarium, and clavicle of the newborn WT and *Irx5* KO mice generated through zygote injection of CRISPR-Cas9. (E) μCT analysis of distal femurs from 4-week-old WT and *Irx5* KO mice. Representative images of three-dimensional reconstruction of μCT. (F) BV/TV of *Irx5* KO mice and control littermates. (G) Tb.N of *Irx5* KO mice and control littermates. (H) Tb.Th of *Irx5* KO mice and control littermates. (I) C.Th of *Irx5* KO mice and control littermates. ***P < 0.001; **P < 0.01; *P < 0.05 versus WT. n.s., *P* > 0.05. Data associated with this figure can be found in S1 Data. BV/TV, bone volume per tissue volume; Cas9, CRISPR-associated protein 9; CRISPR, clustered regularly interspaced palindromic repeats; C.Th, cortical thickness; KO, knockout; n.s., no significant difference; P0, postnatal day 0; sgRNA, single guide RNA; Tb.N, trabecular number; Tb.Th, trabecular thickness; WT, wild type; μCT, microcomputed tomography.

To validate the functions of candidate genes identified from the library screening, we generated individual gene knockouts in mice for *Rln1* and *Irx5* by zygote injection of CRISPR-Cas9. *Rln1*^*–/–*^mice with homozygous frameshift mutations were derived by crossing *Rln1*^*+/–*^mice (Fig 3A and S3A Fig). Newborn mutant pups showed small size and growth retardation compared with the wild-type littermates (Fig 3B). Interestingly, *Rln1*^*–/–*^mice grew normally into adults. Microcomputed tomography (μCT) scanning indicated that the bone volume slightly decreased in *Rln1*^*−/−*^ mice (Fig 3C), suggesting that *Rln1* is involved in prenatal bone development but has less of an effect on bone remodeling. We also generated 2 *Irx5*^−/−^ mouse lines (Fig 3A and S3B Fig) and found that both postnatal and adult *Irx5*^−/−^ mice showed overt skeletal abnormalities (Fig 3D; S3C Fig; S8 and S9 Tables). We thus selected *Irx5*^−/−^ mice for further analyses in this study. Taken together, these data demonstrate the effectiveness and reliability of our ICAHCI-mediated strategy for in vivo functional screening.

### *Irx5* is required for osteoblastogenesis

We then carefully analyzed the bone phenotypes by strictly comparing mutant and wild-type littermates of the same sex obtained by crossing *Irx5*^+/−^ males with females. Bone staining analysis showed significant reduction in body length and delayed clavicular and calvarial mineralization in newborn *Irx5*^−/−^ mice (Fig 3D). All *Irx5*^−/−^ mice survived, grew normally into adults, and were fertile, consistent with previous observations [49, 53]. The μCT analysis revealed that *Irx5* knockout mice had significantly decreased trabecular bone volume in the femur metaphysis (Fig 3E and 3F; S3C and S3D Fig). Meanwhile, both trabecular bone number and thickness decreased significantly (Fig 3G and 3H; S3E and S3F Fig). Moreover, cortical bone thickness also decreased in the knockout mice (Fig 3I and S3G Fig). Taken together, these data suggest that bone mass was reduced in *Irx5* knockout mice.

**Fig 4:**
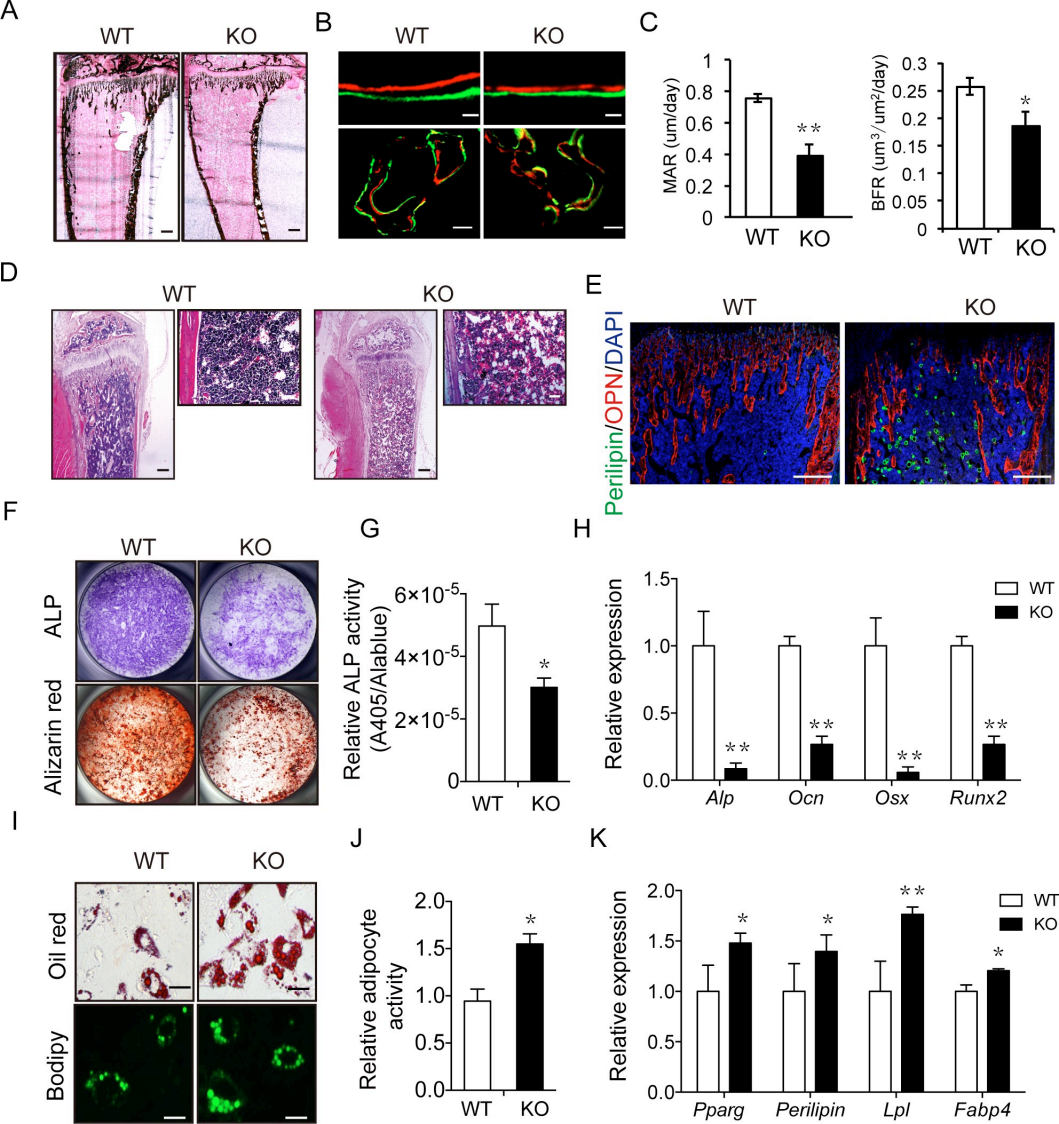
*Irx5* is required for osteogenesis and adipogenesis. (A) von Kossa staining of tibia from 4-week-old WT and *Irx5* KO mice. Scale bar, 300 μm. (B–C) Calcein-alizarin red double labeling of tibiae from 4-week-old WT and *Irx5* KO mice. Representative images visualized by fluorescent microscopy of periosteal bone and trabecular bone (B). Scale bars, 30 μm (top) and 100 μm (bottom). MAR and BFR (C). Data are presented as mean ± SD, *n* = 4 in each group. (D) HE staining of tibiae from 4-week-old WT and *Irx5* KO mice. Scale bars, 300 μm (left) and 100 μm (right). (E) Immunostaining of Perilipin A/B (green) and OPN (red) of tibiae from 4-week-old WT and *Irx5* KO mice. Scale bar, 300 μm. (F) ALP staining of osteoblasts cultured with osteoblast differentiation medium for 7 days, and bone nodule formation visualized by alizarin red staining of osteoblasts cultured for 21 days. (G) Statistical analysis of ALP activity and Alamar Blue activity in the osteoblast differentiation culture via colorimetric readout (A405) and fluorescencent readout, respectively. Data are presented as mean ± SD, *n* = 4 in each group. (H) Gene expression analysis of osteoblast cultured for 4 days was examined by qRT-PCR. Data are presented as mean ± SD, *n* = 4 in each group. (I) Oil Red and Bodipy staining of adipocytes cultured with adipocyte differentiation medium for 6 days. Scale bar, 10 μm. (J) Statistical analysis of percentage of Oil Red positive area via Image J. Data are presented as mean ± SD, *n* = 4 in each group. (K) Gene expression analysis of adipocyte cultured for 6 days was examined by qPCR. Data are presented as mean ± SD, *n* = 3 in each group. **P < 0.01; *P < 0.05 versus WT. Data associated with this figure can be found in S1 Data. ALP, alkaline phosphatase; BFR, bone formation rate; DAPI, 4ʹ,6-diamidino-2-phenylindole; HE, hematoxylin–eosin; KO, knockout; MAR, mineral apposition rate; OPN, osteopontin; qPCR, quantitative polymerase chain reaction; qRT-PCR, quantitative reverse transcription polymerase chain reaction; WT, wild type.

Due to the involvement of both osteoblast-mediated bone formation and osteoclast-mediated bone resorption in bone remodeling, we next attempted to determine which process has been impaired in *Irx5* knockout mice. The von Kossa staining showed an obvious decline in mineral apposition in the *Irx5*^−/−^ tibiae (Fig 4A) and vertebra (S4A and S4B Fig). Meanwhile, double labeling using calcein and alizarin red showed decreased bone formation rate (BFR) in both tibia and vertebra of *Irx5* knockout mice (Fig 4B and 4C; S4C Fig). Moreover, immunostaining demonstrated reduced expression of osteoblastic markers including osteopontin (OPN) (Fig 4E), and osteocalcin (OCN) (S4D Fig) in the trabecular bone region. Together, these results demonstrated decreased osteoblast-mediated bone formation in *Irx5* knockout mice.

We next asked whether abnormal osteoclast activity was also involved in the bone mass reduction in *Irx5* knockout mice. For this, we first analyzed the activity of osteoclasts by staining tartrate-resistant acid phosphatase (TRAP) that typify the osteoclast lineage. The results showed no obvious difference between *Irx5* knockout mice and wild-type controls (S4E and S4F Fig). We next performed in vitro osteoclastogenesis by differentiation of the bone marrow cells in the presence of macrophage colony-stimulating factor (M-CSF) and receptor activator of nuclear factor kappa-B ligand (RANKL) and found similar differentiation potential for *Irx5*^−/−^ bone marrow cells compared with wild-type cells (S4G and S4H Fig), demonstrating normal intrinsic osteoclast differentiation ability of the *Irx5*^−/−^ bone marrow cells. Furthermore, we cocultured wild-type/*Irx5*^−/−^ osteoblast progenitors with wild-type osteoclasts and found that *Irx5*^−/−^ osteoblastogenesis did not affect osteoclasts (S4I and S4J Fig), excluding the possibility of indirect effects of osteoblasts on osteoclasts. Taken together, these results indicate that the decreased bone volume of *Irx5* knockout mice is due to impeded bone formation but not increased bone resorption.

### *Irx5* is involved in bone marrow adipogenesis

Besides analyzing the bone mass of *Irx5* knockout mice, we also analyzed the adipocytes in bone marrow by hematoxylin–eosin (HE) staining to test for an inverse relationship between osteoblastogenesis and adipogenesis in bone marrow cells [54]. As shown in Fig 4D, *Irx5* knockout mice displayed significantly increased numbers of bone marrow adipocytes compared with wild-type littermates. Consistently, immunofluorescent staining of perilipin A, a mature adipocyte marker, also confirmed increased adipogenesis in *Irx5*^−/−^ mice (Fig 4E). Nevertheless, *Irx5* knockout mice had normal ratio of epididymal fat and brown fat mass relative to body weight (S4K–S4M Fig). To address the role of *Irx5* in bone marrow adipogenesis, we analyzed the expression pattern of *Irx5* during osteoblastogenesis and adipogenesis of bone marrow cells by performing quantitative polymerase chain reaction (qPCR). The results showed that the expression of *Irx5* increased during osteoblast differentiation (S5A Fig) and decreased during adipocyte differentiation from the bone marrow mesenchymal stem cells (BMSCs; S5B Fig). Taken together, these data suggest that *Irx5* may be a potential molecular switch in osteoblastogenesis and adipogenesis of bone marrow cells.

### IRX5 promotes osteogenesis and inhibits adipogenesis by suppressing PPARγ activation

We next examined the roles of IRX5 in bone marrow osteoblastogenesis and adipogenesis by inducing *Irx5*^*−/−*^ BMSCs to differentiate into osteoblast and adipocyte in vitro, respectively. Consistent with the in vivo behaviors, *Irx5*^*−/−*^ BMSCs showed decreased osteoblast differentiation ability as shown by reduced alkaline phosphatase (ALP) activity (Fig 4F and 4G) and decreased mineralization nodules through alizarin red histochemical staining (Fig 4F). Meanwhile, the expression levels of osteoblastic markers decreased in differentiated cells (Fig 4H). Inversely, *Irx5*^*−/−*^ BMSCs exhibited higher adipocyte differentiation potency compared with wild-type BMSCs, as demonstrated by an increase in numbers of adipocytes via Oil Red and Bodipy staining (Fig 4I and 4J) and improved expression levels of adipocyte markers in cultured cells via qPCR (Fig 4K). Taken together, these data demonstrate that *Irx5* promotes osteoblast differentiation and inhibits adipocyte differentiation during bone development and remodeling.

**Fig 5:**
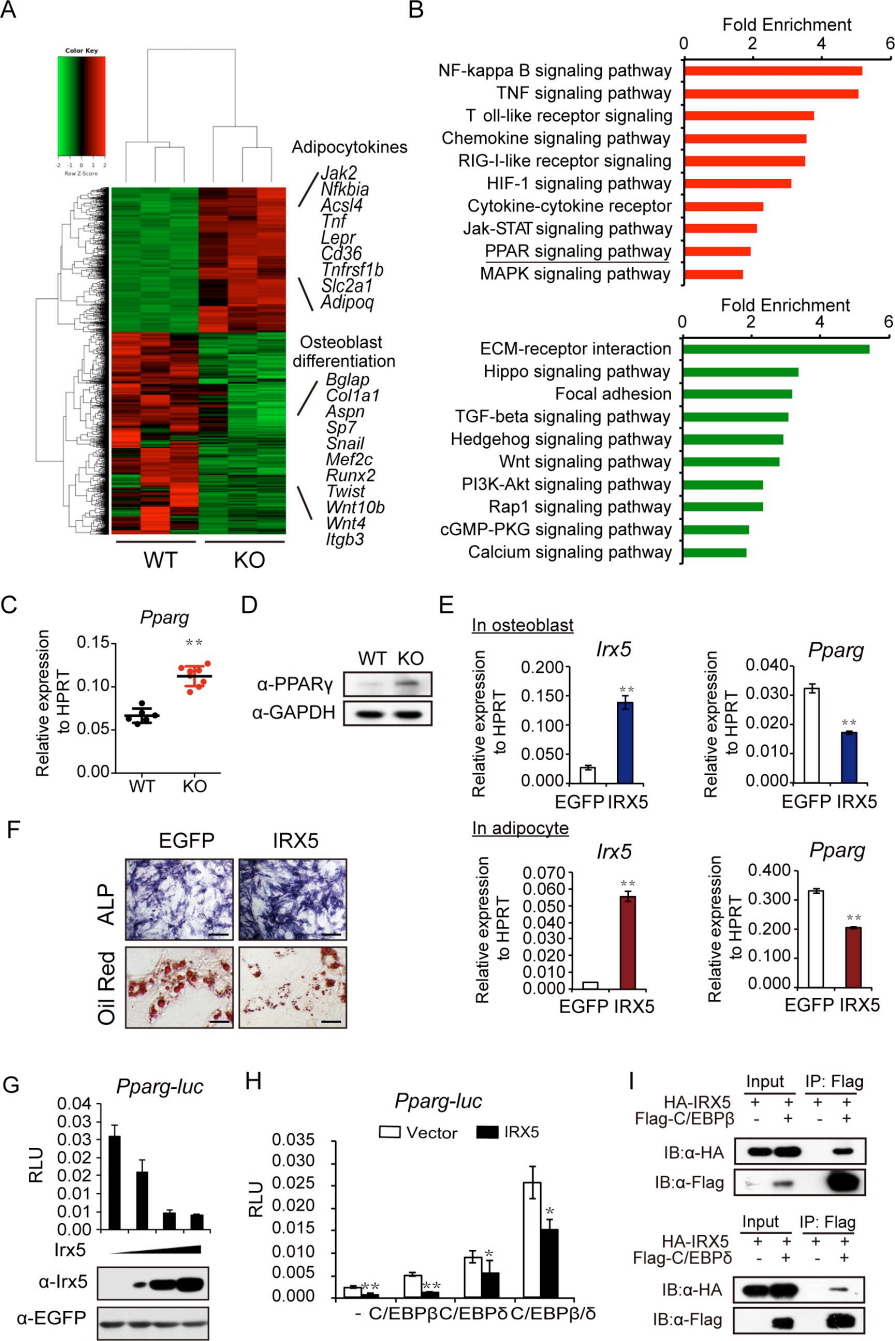
*Irx5* promotes osteogenesis and inhibits adipogenesis by suppressing PPARγ activation. (A) Heat map of RNA-seq data between wild-type and *Irx5* knockout BMSCs cultured in osteoblast differentiation medium for 4 days, *n* = 3 for each group. (B) Up-regulated (red) and down-regulated (green) pathways associated with significantly regulated genes (*P* < 0.05) in *Irx5* knockout versus wild-type control. (C) qPCR results of *Pparg* expression in the tibiae and femurs from wild-type and *Irx5* knockout mice. *n* = 6 for WT and *n* = 8 for KO mice. (D) Western blotting analysis of PPARγ in the tibiae and femurs from wild-type and *Irx5* knockout bone tissue. (E) Gene expression levels of *Irx5* and *Pparg* in the cultured osteoblast and adipocyte from wild-type BMSCs infected with lentivirus expressing vector control or IRX5. (F) ALP staining of the cultured osteoblast and Oil Red staining of the cultured adipocyte from wild-type BMSCs infected with lentivirus expressing vector control or IRX5. Scale bars, 50 μm (top) and 10 μm (bottom). (G) *Irx5* inhibits luciferase activity as judged by *Pparg-luc* reporter activity in C3H10T1/2 cells. (H) *Irx5* represses the transactivation ability of CEBPβ and CEBPδ in inducing *Pparg-luc* activity in C3H10T1/2 cells. *n* = 3. (I) In vitro coimmunoprecipitation of HA-IRX5 with Flag-CEBPβ or Flag-CEBPδ following transfection into 293FT cells. **P < 0.01; *P < 0.05 versus control. Data associated with this figure can be found in S1 Data. ALP, alkaline phosphatase; BMSC, bone marrow mesenchymal stem cell; CEBPβ, CCAAT/enhancer binding protein beta; CEBPδ, CCAAT/enhancer binding protein delta; cGMP-PKG, cGMP-dependent protein kinase; C3H10T1/2, murine mesenchymal stem cell line; ECM, extracellular matrix; EGFP, enhanced green fluorescent protein; GAPDH, glyceraldehyde-3-phosphate dehydrogenase; HA, hemagglutinin; HIF-1, hypoxia-inducible factor 1; HPRT, hypoxanthine guanine phosphoribosyl transferase; IB, immunoblotting; IP, Immunoprecipitation; IRX5, iroquois homeobox 5; Jak-STAT, Janus kinase/signal transducers and activators of transcription; KO, knockout; MAPK, Mitogen-activated protein kinases; NF, nuclear factor; PI3K-AKT, phosphatidylinositol 3-kinase- Protein Kinase B; PPARγ, peroxisome proliferator activated receptor γ; *Pparg-luc*, *Pparg* promoter-luciferase; qPCR, quantitative polymerase chain reaction; Rap1, Ras-proximate-1; RIG-I, retinoic acid-inducible gene I; RLU, relative light unit; RNA-seq, RNA sequencing; TGF, transforming growth factor; TNF, tumor necrosis factor; Wnt, Wingless/Integrated; WT, wild type; 293FT, human embryonic kidney cell line.

To explore the downstream molecules regulated by *Irx5*, we performed RNA sequencing (RNA-seq) analysis and compared the gene expression profiles of wild-type and *Irx5*^−/−^ BMSCs (4 days post osteoblast differentiation; Fig 5A). Gene set enrichment analysis (GSEA) was then employed to identify significantly enriched gene ontology (GO) terms. The results showed that osteoblast differentiation and developmental regulators were significantly down-regulated, and the adipocyte differentiation regulators and marker genes were up-regulated in the *Irx5* knockout cells (Fig 5B). We confirmed the decreased levels of osteoblast marker genes—including *Alp*, *Runx2*, *Osx*, *Col1a1*, and *Ocn* (S5C Fig)—and increased adipocyte marker genes—including *Tnf*, *Acsl5*, *Gyk*, *Lpl*, and *Adipoq* (S5D Fig)—in the *Irx5* knockout BMSCs by qPCR. Interestingly, Kyoto Encyclopedia of Genes and Genomes (KEGG) pathway analysis indicated that the PPARγ signaling pathway was up-regulated in the knockout cells (Fig 5B). The transcription factor PPARγ plays a critical role in bone development by inducing adipogenesis and inhibiting osteoblastogenesis [54]. We thus focused on the role of IRX5 in the regulation of the PPARγ signaling pathway.

The up-regulation of PPARγ in the *Irx5*^*−/*−^ long bones was confirmed at both mRNA and protein levels (Fig 5C and 5D). Consistently, the known PPARγ downstream targets, including *Gyk*, *Lpl*, and *Tnf* [55], were increased in the *Irx5*^*−/−*^ long bones (S5E Fig). These results suggest that IRX5 may act mainly as a transcriptional repressor during bone development, consistent with the previous studies demonstrating the role of IRX5 as a repressor in the development of other organs [53, 56]. To further confirm that PPARγ is the downstream factor of IRX5, we attempted to reverse the osteoblast and adipocyte defects during differentiation from *Irx5*^*−/−*^ BMSCs through the knockdown of the *Pparg* using short hairpin RNAs (shRNAs). All designed shRNAs efficiently reduced the expression levels of *Pparg* in wild-type and knockout BMSCs (S6A Fig). As expected, knockdown of *Pparg* in *Irx5* knockout BMSCs could rescue the decreased osteoblast differentiation and increased adipocyte differentiation compared with the wild-type cells (S6B–S6G Fig). We overexpressed *Irx5* in the mouse primary BMSCs to directly investigate the role of *Irx5* in regulating *Pparg* expression, followed by differentiation into osteoblast and adipocyte. Consistently, *Irx5* overexpression significantly repressed *Pparg* expression (Fig 5G), leading to increased osteoblast differentiation and decreased adipocyte differentiation (Fig 5E and 5F).

Finally, to demonstrate that IRX5 transcriptionally represses *Pparg*, we used luciferase reporter assay and found that IRX5 reduced the baseline activity of *Pparg* promoter (Fig 5H). Since the CCAAT/enhancer binding protein beta/delta (C/EBPβ/δ)- CCAAT/enhancer binding protein alpha (C/EBPα)-PPARγ transcriptional cascade has been extensively studied in adipocyte differentiation [57, 58], we then analyzed the relationship between IRX5 and C/EBPβ/δ and found that IRX5 repressed the activation of *Pparg* promoter through direct interaction with C/EBPβ and C/EBPδ (Fig 5I). Our results show that the IRX5 attenuated *Pparg* expression through binding to C/EBPβ/δ complex. Taken together, these data suggest that *Irx5* promotes osteogenesis and inhibits adipogenesis by suppressing PPARγ activation.

## Discussion

**Fig 6:**
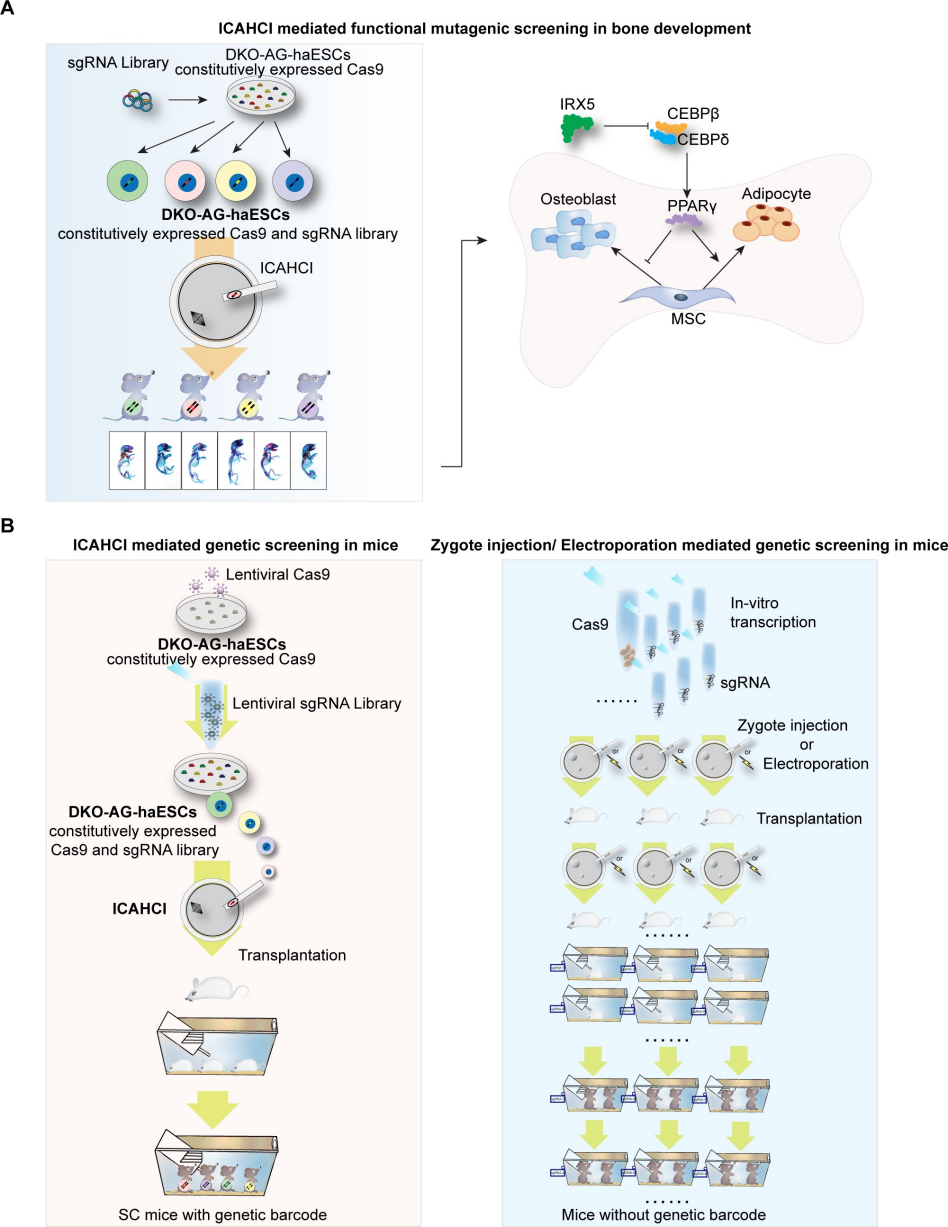
Schematic for ICAHCI-mediated genetic screening in mice via CRISPR/Cas9. (A) Schematic for ICAHCI-mediated functional mutagenic screening in bone development in mice via CRISPR/Cas9. (B) Schematic for comparison of ICAHCI and zygote injection/electroporation mediated genetic screening in mice via CRISPR/Cas9. Cas9, CRISPR-associated protein 9; CEBPβ, CCAAT/enhancer binding protein beta; CEBPδ, CCAAT/enhancer binding protein delta; CRISPR, clustered regularly interspaced palindromic repeats; DKO-AG-haESC, double knockout androgenetic haploid embryonic stem cell; ICAHCI, intracytoplasmic AG-haESCs injection; IRX5, iroquois homeobox 5; MSC, mesenchymal stem cell; PPARγ, peroxisome proliferator activated receptor γ; sgRNA.

In this study, we provide the first evidence to show that ICAHCI technology combined with CRISPR-Cas9 could be a powerful method to identify pivotal genes that are involved in a developmental process from a medium-scale gene library in mice (Fig 6A). Our approach has a number of advantages over existing technologies such as direct injection or electroporation of CRISPR-Cas9 into zygotes for in vivo genetic screening (Fig 6B and S10 Table) [59–65]. First, DKO-AG-haESCs carrying constitutively expressed Cas9 and sgRNA library provide a unique system in which Cas9 and sgRNAs targeting different genes can be delivered into oocytes through ICAHCI, ensuring the generation of SC pups with different mutations in one step and avoiding the individual preparation of sgRNAs and/or RNPs and Cas9 mRNA for zygote injection or electroporation. Second, the mutant gene in SC pups can be easily identified from the injected haploid cell by amplifying the integrated sgRNA (just like a “barcode” of the injected cell and the resultant SC mouse) [25], enabling maintenance of SC animals with different mutations together and avoiding individually keeping mice from each sgRNA generated through zygote injection or electroporation strategies. Meanwhile, genotyping of mutant SC pups can be done through PCR with 1 pair of common primers for sgRNA, followed by sequencing analyses. In contrast, the genotype of mice generated by zygote injection or electroporation shall be determined by PCR using primers for specific genes. Third, our strategy needs fewer animals. In the current study, a total of 3,601 SC embryos were constructed from 25 ICAHCI experiments and were transferred into around 90 pseudopregnant females (40 SC embryos for each) to obtain 426 pups, which resulted in screening out 4 genes from 72 preselected genes (a total of 216 sgRNAs). However, for electroporation or injection of CRISPR RNPs into zygotes, if we injected around 20 fertilized oocytes for each sgRNA (just considering the minimal number of embryos for transferring into one recipient mouse), we would need a total of 4,320 zygotes for at least 216 independent zygotic injection or electroporation experiments. Meanwhile, at least 216 recipient pseudopregnant mice were needed for embryo transplantation (1 for each sgRNA). Fourth, although the developmental potential of SC embryos is lower than that of fertilized embryos that are used for zygote injection or electroporation, our strategy has comparable efficiency of the biallelic mutant mouse derivation to that of other strategies (S11 Table) [60–65]. In addition, our strategy for the loss-of-function screen in mice enables generation of a large amount of sex-matched SC mice (females) carrying different biallelic mutant genes in one step and phenotypic analysis at the same age, thus conforming to the 2 important requirements for the phenotypic analysis of bone during development [66, 67].

In this study, 4 genes associated with bone development, including *Zic1*, *Clec11a*, *Rln1*, and *Irx5*, were identified from a total of 426 SC pups generated from a haploid cell line with an sgRNA library targeting 72 preselected genes. Although 72 genes were targeted in 426 mice at different frequencies, we only performed genotyping analyses of the pups (score ≥ 2) carrying a gene that were targeted twice. This strict standard of phenotype-driven screening is less labor intensive and expensive and ensures the accuracy of screening results; however, it may increase the possibility of missing some important genes. Nevertheless, 4 identified genes are indeed involved in bone development as demonstrated by the generation of mouse models carrying individual mutation or earlier work [42, 47], showing the effectiveness and efficiency of our strategy.

In addition to *Zic1* and *Clec11a* that have been demonstrated previously to be involved in bone development [42, 47], we proved that 2 well-studied genes, *Rln1* and *Irx5*, are involved in bone development. *Rln1*, identified 2 decades ago [68], is a protein hormone belonging to the insulin superfamily and displays important roles in human reproduction as well as collagen synthesis and angiogenesis [69]. A recent study has shown its function in osteoblastic differentiation in cultured cells [48]. Interestingly, we showed that the newborn *Rln1* mutant mice exhibited reduced size, whereas adult mutant mice (12 weeks old) showed normal bone density, implying that *Rln1* plays an important role during prenatal bone development, and the defects may be rescued during bone remodeling through functional redundancy of other family genes [70]. Previous studies based on *Irx5*^−/−^ mice have demonstrated that *Irx5* is necessary for retinal cone bipolar cell development [52] and cardiac repolarization gradient [53]. However, the defects in bone development have not been observed in *Irx5*^−/−^ mice, although *IRX5* mutation is related to human craniofacial development defects [50]. Utilizing our screening system that enables analysis of bone phenotypes by parallelly comparing mutant mice with same sex and age, we discovered that *Irx5* is involved in bone development, providing the direct evidence to support that *IRX5* mutation accounts for craniofacial abnormalities. In summary, our DKO-AG-haESCs carrying an sgRNA library via ICHACI technology open new avenues for targeted genetic screening at the organism level, especially for developmental processes in mice.

## Materials and methods

### Ethics statement

All animal procedures were performed under the ethical guidelines approved by the Animal Care and Use Committee of Shanghai Institute of Biochemistry and Cell Biology, Chinese Academy of Sciences (approval number: IBCB0070).

### DKO-AG-haESCs culture

Detailed haploid cell culture and ICAHCI protocol has been previously described [23, 24]. Mouse DKO-AG-haESCs (*IG*^ΔDMR^-*H19*^ΔDMR^-AGH-2 cell line) were cultured in ESC medium supplemented with 1,500 U/ml LIF (Millipore, USA), 3 μM CHIR99021 (Selleck, USA), and 1 μM PD0325901 (Selleck, USA). For haploid cell sorting, DKO-AG-haESCs were dissociated using 0.05% trypsin-EDTA (1×; Gibco, USA), washed with DPBS (Gibco, USA), and stained in 15 μg/ml Hoechst 33342 (Molecular Probes, USA) for 5 minutes in a 37°C water bath. Subsequently, haploid cells were enriched using flow cytometry (FACS AriaII, BD Biosciences, USA) according to the haploid 1C peak.

### Generation of SC mice via ICAHCI

For ICAHCI, DKO-AG-haESCs arrested at M phase by culturing in ESC medium containing 0.05 μg/ml demecolcine (Sigma-Aldrich, USA) for 8 hours were used for intracytoplasmic injection. The synchronized AG-haESCs were trypsinized, washed 3 times with HEPES-CZB medium, and suspended in HEPES-CZB medium containing 3% (w/v) polyvinylpyrrolidone. Each nucleus from M-phase haploid cells was injected into an MII-arrested oocyte using a Piezo-drill micromanipulator. The reconstructed oocytes were cultured in CZB medium for 1 hour and then activated for 5 to 6 hours in activation medium without CB. Following activation, all of the reconstructed embryos were cultured in EmbryoMax KSOM Medium (Sigma-Aldrich, MR-106-D, USA) at 37°C under 5% CO_2_ in air until the two-cell stage. For embryo transplantation, 17 to 20 reconstructed two-cell embryos were transferred into each oviduct of a pseudopregnant ICR female at 0.5 days post coitum (dpc). SC pups were naturally delivered or removed from the uteri of their euthanized recipient mothers at 19.5 dpc. After cleaning fluid from their air passages, the pups were kept in a warm box supplied with oxygen.

### Generation of mutant mice via zygote injection

For Cas9 mRNA or sgRNA transcription, the T7 promoter sequence was added to the Cas9 coding region or the sgRNA template by PCR amplification using primers shown in S12 Table. In vitro transcription of Cas9 mRNA was done using mMESSAGE mMACHINE T7 ULTRA kit (ThermoFisher Scientific, AM1345, USA) and sgRNAs targeting *Irx5* and *Rln1* using MEGAshortscript T7 kit (ThermoFisher Scientific, AM1354, USA), according to the manufacturer’s instructions. Both Cas9 mRNA and sgRNAs were then purified using MEGAclear Transcription Clean-Up Kit (ThermoFisher Scientific, AM1908, USA) and stored at −80°C.

One-cell embryos were collected from superovulated wild-type C57BL/6J female mice that were mated with wild-type C57BL/6J male mice. Cas9 mRNA (50 ng/μl) and sgRNA targeting *Irx5* or *Rln1* (50 ng/μl) were mixed together and then coinjected into the one-cell embryos. The injected embryos were cultured in EmbryoMax KSOM Medium (Sigma-Aldrich, MR-106-D, USA) until the two-cell stage and then transferred into oviducts of recipients at 0.5 dpc. Recipient mothers delivered pups at 19.5 dpc.

### Lentiviral sgRNA library construction

SgRNA sequences to each gene were selected from Feng Zhang’s Mouse Genome- scale CRISPR Knock-Out (GeCKO) lentiviral pooled libraries version 2.0 part A and synthesized (S3 Table). Forward and reverse oligonucleotides were mixed at 10 μM each in 10 mM Tris-HCl (pH8.0) and 5 mM MgCl_2_ to a final volume of 50 μL. Each reaction tube of the mixture was placed in a tray of boiling water until it cooled down to room temperature. The annealed oligonucleotides were then mixed and cloned into the Bbs I -digested pKLV-U6gRNA(BbsI)-PGKpuro2ABFP plasmid (Addgene,#-50946). To preserve the diversity and integrity of the library, at least 50-fold coverage of the whole sgRNAs were recovered in each transformation using *Escherichia coli* DH5α competent cells (TaKaRa, Cat#9057, USA). Resulting bacteria were scraped off the plate using a spreader and combined in a centrifuge tube. The plasmid DNA was purified with Plasmid Maxi kit (Tiangen, DP117, China).

### Lentiviral infection of DKO-AG-haESCs

The lentiCas9-Blast plasmid was obtained from Addgene (Addgene, #52962) [41], and the lentiviral sgRNA library was constructed as described above. Lentivirus was produced by the cotransfection of HEK293T cells in a 10-cm dish with 3 μg of a lentiviral vector (lentiCas9-Blast or lenti-sgRNA library) and 9 μg of ViraPower Lentiviral Packaging Mix (ThermoFisher Scientific, K497500, USA) using Lipofectamine 3000 Reagent (ThermoFisher Scientific, L3000015, USA) in accordance with the manufacturer’s manual. The virus-containing supernatant was harvested 72 hours after transfection, concentrated with Lenti-Concent in virus precipitation solution (System Biosciences, EMB810A-1, USA), and then stored at −80°C. The method to determine the volume of virus needed to achieve a multiplicity of infection (MOI) of 0.3 (to ensure that most cells receive single copy of the lentiviral vector) has been described previously by Zhong and colleagues [24]. To generate the DKO-AG-haESC lines carrying constitutively expressed Cas9, first of all, DKO-AG-haESCs in 10-cm dishes were infected in ESC cultural medium with optimal volume of lentiCas9-Blast lentivirus for 48 hours. After selection in culture medium containing blasticin antibiotics for 72 hours, the infected cells were collected and sorted for haploid cells using flow cytometry (FACS AriaII, BD Biosciences, USA) according to the haploid 1C peak. The resulting colonies were picked and further expanded. To generate DKO-AG-haESCs carrying constitutively expressed Cas9 and sgRNA, the established Cas9-expressing DKO-AG-haESC line was first enriched for haploid cells and then infected with lentiviral sgRNA in ESC cultural medium for 48 hours at an MOI of 0.3. After selection in culture medium containing puromycin antibiotics for 48 hours, the infected cells were collected and sorted for haploid cells using flow cytometry (FACS AriaII, BD Biosciences, USA). For DKO-AG-haESC sgRNA library construction, the enriched cells were no less than 1,000× of the total number of sgRNAs. The resulting cells were expanded for genomic DNA extraction and ICAHCI experiments.

### DNA sequencing analysis

For deep sequencing analysis of the Cas9B-3-BD cells carrying Cas9 and an sgRNA library, DNA was purified using the Genomic DNA Kit (Tiangen, DP304-03, China) according to the manufacturer’s instructions and was used as the PCR template. The sgRNA inserts were PCR amplified using the primers shown in S12 Table, and the resulting library was sequenced on a HiSeq4000 (Illumina, USA) with a single-end 50-bp run.

For analysis of mutation efficiency corresponding to each sgRNA-targeting site, the sgRNA integrated in each haESC colony or SC mouse was amplified using the universal primer pair at first. Then, according to sgRNA sequence, primers for each targeting site were designed (S12 Table). Finally, by PCR amplification and the following TA cloning (Takara, 6013, USA), the mutation efficiency was detected by sequencing.

### Off-target effect analysis

According to the predicted off-target sites of the sgRNA targeting *Irx5* by Cong and colleagues’ website (http://crispr.mit.edu/) [71], we choose 12 potential sites that had comparatively higher off-target risk. The sequences of the primers for off-target detection are listed in S12 Table.

### Skeletal whole-mount staining

Mice were euthanized, skinned, and eviscerated. The skeletal samples were dehydrated in 95% ethanol overnight and then in acetone for 24 hours. Skeletons were stained with alcian blue and alizarin red for 2 days as described previously by McLeod [40]. Soft tissue was then cleared in successive solutions of 1% KOH and glycerol.

### μCT analysis

For μCT analysis, femurs isolated from age- and sex-matched mice were fixed in 70% ethanol and scanned using SkyScan 1176 with a spatial resolution of 9 μm. From the scans, a 1.5-mm long region of the distal metaphysis was analyzed. Three-dimensional images were reconstructed using a fixed threshold. Unbiased, three-dimensional microstructural properties of trabecular bone and cortical bone, including the percentage of trabecular bone volume per tissue volume (BV/TV), trabecular thickness (Tb.Th), and trabecular number (Tb.N), were calculated for the trabecular region of the distal femur metaphysis, and cortical thickness (C.Th) was calculated for the cortical bone of the diaphysis of the distal femur using previously described methods by Hildebrand and colleagues [72].

### Histological analysis

Histological analysis were performed as previously described by Han and colleagues [73]. Briefly, for dynamic histomorphometry, mice received intraperitoneal injection of calcein (Sigma, C0875, USA) at a dose of 20 mg/kg body weight and alizarin red (Sigma, A5533) at a dose of 25 mg/kg body weight with an interval of 4 days for injection. Three days after the alizarin red injection, the mice were euthanized, and the tibiae and vertebra were fixed in 4% paraformaldehyde (PFA) for 48 hours at 4°C, dehydrated in ethanol and acetone, and embedded in resin. The samples were then cut into 4-μm thick sections using a Leica RM2265 microtome. Bone mineral apposition rate (MAR) and BFR were measured as previously described by Dempster and colleagues [74].

For paraffin sections, the femurs and tibiae were fixed in 4% PFA and decalcified in 15% EDTA. Samples were dehydrated with gradient ethanol and xylene, embedded in paraffin blocks, and cut into 8-μm–thick sections using a Leica RM2235 microtome. Sections were dewaxed, rehydrated, and stained with HE and TRAP (Sigma, S387A-1KIT, USA) according to the manufacturer’s instructions.

For immunohistochemical staining, the paraffin sections were first dewaxed and rehydrated and then incubated with protease K at 37°C for 30 minutes for antigen retrieval. The sections were blocked and permeabilized with 3% BSA and 0.2% Triton-X100 in PBS at room temperature for 45 minutes and incubated with primary antibodies at 4°C overnight (mouse anti-RUNX2, Santa Cruz, sc-390351, USA], mouse anti-CTSK [Santa Cruz, sc-48353, USA]). After washing, the sections were incubated with the corresponding biotinylated secondary antibodies for 45 minutes at room temperature using a VECTASTAIN ABC kit (Vector Labs, PK-6100, USA) or a mouse-on-mouse (M.O.M.) detection kit (Vector Labs, PK-2200, USA). Diaminobenzidine (DAB; Vector Labs, SK-4100, USA) was used as the substrate. The nuclei were counterstained using hematoxylin, and the sections were dehydrated and mounted using neutral balsam.

For immunofluorescencent staining, the frozen sections were rehydrated with PBS, blocked and permeabilized with 3% BSA and 0.2% Triton-X100 in PBS for 45 minutes at room temperature, and then incubated with the primary antibodies (donkey-anti-OPN [R&D, AF808, 1:500, USA], rabbit-anti-perilipin A/B [Sigma, P1873, 1:1,000, USA]) at 4°C overnight. The sections were incubated with the fluorescence labeling secondary antibodies (donkey-anti-rabbit Alexa Fluor488 [Molecular Probes, 1:1,000, USA], donkey-anti-goat Cy3 [Molecular Probes, 1:1,000], USA) for 1 hour at room temperature after washing. Nuclei were counterstained with 4ʹ,6-diamidino-2-phenylindole (DAPI; Sigma, D9542, USA). Sections were mounted using fluorescence mounting medium (Dako, S3023, USA).

### RNA extraction and quantitative RT-PCR analysis

Total RNA was extracted using TRIzol (Sigma, T9424, USA) following the standard protocol. RNA was reverse transcribed into cDNA using TaKaRa PrimeScript Reverse Transcriptase (TaKaRa, RR037A, USA). qPCR was performed on a Bio-Rad CFX96 Real-Time PCR Detection System (Bio-Rad Laboratories, USA) using SYBR green (Life technologies, S7563, USA). The primer sequences for qPCR are listed in S12 Table.

### Protein extraction and immunoblotting

Total proteins were extracted from bone tissues crushed with liquid nitrogen and lysed in tissue lysis buffer (50 mM Tris [pH 7.4], 150 mM NaCl, 1% TritonX-100, 1 mM EDTA, and 0.1% SDS) supplemented with protease inhibitors cocktail (Sigma, USA). Lysates containing 30 μg of protein were separated by 10% SDS-polyacrylamide gel electrophoresis (PAGE), followed by western blotting according to the standard protocol. The following primary antibodies were used: IRX5 (Santa Cruz, sc-98397, 1:1,000, USA), PPARγ (Santa Cruz, sc-7273, 1:1,000, USA), HA probe (Santa Cruz, sc-9372, 1:5,000, USA), FLAG (Sigma, F3165, USA), HRP conjugated-rabbit-anti-mouse (Dako, P0260, 1:5,000, USA), and HRP conjugated-swine-anti-rabbit (Dako, P0217, 1:5,000, USA).

### Isolation and culture of bone marrow cells

Femurs and tibiae were cleaned thoroughly, and the epiphysis and metaphysis were then cut off. The bone marrow was flushed out with DPBS using a 0.45 mm needle. The cell suspension was passed through a 70 μm nylon mesh (BD Falcon, BD Biosciences, 352350, USA) and plated in a 6-well plate in growth medium (α-MEM containing 10% fetal bovine serum [FBS; Ausbian, VS500T, Australia] and 1% penicillin/streptomycin [Gibco, 10378–016, USA]) and were passaged at 80% to 90% confluence.

### In vitro differentiation assays

The cell culture and in vitro differentiation assay were performed as previously described by Han and colleagues [73]. Briefly, for osteoblast differentiation, 2 × 10^5^ primary bone marrow cells were plated into each well of a 96-well plate and cultured in osteoblast induction medium (α-MEM containing 10% FBS, 5 mM β-glycerophosphate [Sigma, G9422, USA], 50 μg/ml L-ascorbic acid [Sigma, A5960, USA], and 1% penicillin/streptomycin). The media were changed every 3 days. After 7 days of induction, cells were fixed in 10% neutral buffered formalin (Sigma, HT501320, USA) and stained with BCIP/NBT ALP staining kit (Beyotime, C3206, China) following the manufacturer’s instructions. For quantitative analysis of cell growth and ALP activity, cells cultured for 4, 7, and 10 days were incubated with Alamar Blue (Thermo, 88951, USA) for 2 hours at 37°C, and the fluorescence was read with a multimode plate reader (Envision, Perkin Elmer) at 560 nm excitation wavelength and 590 nm emission wavelength following the manufacturer’s instructions. Cells were then washed with PBS, incubated with ALP substrate solution (1 mg/ml phosphatase substrate [Sigma, S0942, USA]) for 20 minutes, and read in an EnVision plate reader at an absorption wavelength of 405 nm.

For adipocyte differentiation, 6 × 10^5^ cells/ml were plated in each well of a 96-well plate and cultured with adipocyte induction medium (α-MEM with 10% FBS, 50 mM dexamethasone [Sigma, D1756, USA], 100 nM rosiglitazone [Sigma, R2408, USA], 500 nM 3-isobutyl-1-methylxanthine [IBMX; Sigma, I5879, USA], 10 mg/ml insulin [Sigma, 91077c, USA], and 1% penicillin/streptomycin) for 48 hours followed by adipocyte maintenance medium (α-MEM with 10% FBS, 10 mg/ml insulin, and 1% penicillin/streptomycin) for 24 hours. Cells were then fixed with 4% PFA for 10 minutes and stained with 2 mg/ml Oil Red O (Sigma, O1391, USA) or Bodipy 493/503 (Molecluar Probes, D3922, USA).

For osteoclast differentiation, bone marrow-derived macrophages (BMMs) were isolated from the femur and tibia of mice as described previously by Dai and colleagues [75]. The nonadherent cells were seeded at a density of 3 × 10^6^ cells/ml in the 96-well-plate BMM induction medium (α-MEM supplemented with 10% FBS, 10 ng/ml M-CSF, and 1% penicillin/streptomycin) and cultured for 3 days. Furthermore, media were changed to osteoclast induction medium (α-MEM supplemented with 10% FBS, 20 ng/mL M-CSF, 150 ng/ml RANKL, and 1% penicillin/streptomycin) and cultured for 4 days. The cultured supernatant was harvested for detection of TRAP activity. A TRAP stain of the cell was performed using a TRAP staining kit (Sigma, S387A-1KIT, USA) following the manufacturer’s instructions. The quantification of TRAP activity was performed following the method previously described by Dai and colleagues [75]. Briefly, the cultured supernatant collected was incubated with 0.33 M tartrate solution containing phosphatase substrate (Sigma, P4744, USA) at 37°C for 2 hours and stopped with 3 N NaOH. TRAP activity was measured at the maximum wavelength of 405 nm in an EnVision plate reader.

For osteoblast and osteoclast coculture assay, an equal number of mouse osteoblast progenitor cells from calvaria of neonatal wild-type/*Irx5*^−/−^ knockout mice were seeded into wells of a 24-well plate with α-MEM containing 10% FBS. Bone marrow cells isolated from wild-type mice were cultured for 24 hours, and then the nonadherent BMM cells were collected and cultured with the osteoblast progenitors in the presence of 1,25-dihydroxyvitamin D3 (10 nM; Sigma-Aldrich, USA) and PGE2 (1 uM; Sigma-Aldrich, USA) for 6 days. Osteoclast formation was identified by TRAP staining and by determining TRAP activity in the supernatant media.

### Immunoprecipitation

For coimmunoprecipitation, lysates were incubated with Anti-FLAG M2 Affinity Gel (Sigma, F2426, USA) at 4°C overnight. Precipitates were washed with lysis buffer, separated by SDS-PAGE, and immunoblotted with indicated antibodies.

### Luciferase assay

C3H10 T1/2 cells were transfected with a PPARG promoter-driven luciferase reporter plasmid and varied combinations of IRX5, CEBPβ, and CEBPδ expression constructs [76] using Effectene transfection reagent (Qiagen, USA) following the manufacturer’s instructions. Luciferase assays were performed 48 hours after transfection using the Dual-Luciferase Reporter Assay System (Promega, USA) following the manufacturer’s instructions.

### Quantification and statistical analysis

Data generated from independently obtained data sets are represented as mean ± SD. *n* values represent the number of separate biological replicates. Statistical significance was determined using paired or unpaired two-tailed Student *t* test or one-way ANOVA with Tukey’s multiple comparison analysis as appropriate. *P* < 0.05 was considered to be statistically significant (*P < 0.05, **P < 0.01, ***P < 0.001).

### Multinomial distribution analysis

There are a total of *m* different types of specific genes in a population of cells, and each cell includes only 1 specific gene. Assume the following 2 conditions:

1. The rate or probability of gene type-i in the population is *p*_*i*_, I = 1,…,*m* with $\sum_{i=1}^{m}p_{i}=1$;

2. Randomly sample *n* cells from the population.

The process to choose *n* cells can be described as a random variable *Y* = (*x*_1_,…,*x*_*m*_), where *x*_1_,*x*_2_,…,*x*_*m*_ are *m* independent random variables. *n*_i_ is the number of cells with the gene type-i. Thus, *x*_*i*_ = *n*_*i*_ means *n*_i_ cells with the gene type-i are chosen for i = 1,…,*m*. Clearly, *n*_i_ is less than or equal to *n*. Then, the probability *p*(*Y*≥*I*_*m*_) follows the multinomial distribution

$$p(Y\geq I_{m})==\sum_{n_{1},\cdots ,n_{m}}\frac{n!}{n_{1}!\cdots n_{m}!}p_{1}^{n_{1}}.p_{2}^{n_{2}}\cdots p_{m}^{n_{m}},$$ (1)

where *n*_*i*_≥1, i = 1,…,*m* and *I*_*m*_ = (1,…,1) is *m*-dimensional vector. Clearly, Eq 1 is the probability that *n* cells cover all types of genes.

To numerically calculate the probability *p*(*Y*≥*I*_*m*_), we use Monte Carlo method to approximately estimate the probability. The algorithm can be stated as follows.

**Algorithm 1: Estimating the probability by Monte Carlo method**

Step 1. We randomly choose *n* cells. Then we have the numbers of gene types as *x*_1_ = *n*_1_,*x*_2_ = *n*_2_,…,*x*_*m*_ = *n*_*m*_ by counting the numbers of gene types among *n* cells. All of those numbers are represented by Y^k^, where k means the iteration-k.

Step 2. Repeat Step-1 for K times, where *k* = 1,…,*K*. Then, we have *Y*^1^,…,*Y*^*K*^.

Step 3. By sampling K times, we can obtain $\sum_{k=1}^{K}I_{E_{n}}(Y^{k})$, where $E_{n}=\{J_{m}|m_{0}\leq \sum_{i=1}^{m}I(j_{i}\neq 0)\leq m;\sum_{i=1}^{m}j_{i}=n;J_{m}=(j_{1},j_{2},\ldots ,j_{m})\}$, to estimate the probability that these *n* cells cover at least *m*_0_ (*m*_0_ less than or equal to *m*) types of genes. It can be easily proven that $\sum_{k=1}^{K}I_{E_{n}}(Y^{k})$ is the unbiased estimation of *p*(*Y*∈*E*_*n*_), especially, when $m_{0}=m,E_{n}=\{J_{m}|J_{m}\geq I_{m};\sum_{i=1}^{m}j_{i}=n;J_{m}=(j_{1},j_{2},\ldots ,j_{m})\}$, $\sum_{k=1}^{K}I_{E}(Y^{k})=\sum_{k=1}^{K}I(Y^{k}\geq I_{m})/K$

**Algorithm 2: Estimating the numbers by Monte Carlo method**

Step 1. We give a series of *n*, where, *n* = 300,400,500,…,1200.

Step 2. For each *n*, we repeat Step-1, Step-2, and Step-3 of Algorithm-1. And then we have $\sum_{k=1}^{K}I_{E_{n}}(Y^{k})$, and the estimation of *p*(*Y*∈*E*_*n*_) for each *n*.

Step 3. For a given probability p*, we choose the minimal *n* such that $\sum_{k=1}^{K}I_{E_{n}}(Y^{k})$ is more than p*.

Thus, based on the above 2 algorithms, we can obtain the minimal number of cells that can cover at least *m*_0_ types of specific genes with more than the given probability p*.

## Supporting information

S1 Fig DKO-AG-haESCs carrying constitutively expressed Cas9 and an sgRNA support the efficient generation of biallelic mutant SC mice with relevant phenotype in one step. (A–B) Schematic of the Cas9/sgRNA-targeting sites in *Trp53* (A) and *Fgf10* (B). The sgRNA-targeting sequence is underlined, and the PAM sequence is labeled in green. (C) Phase-contrast image (left) and flow analysis (right) of Cas9B-3, a DKO-AG-haESC line carrying constitutively expressed Cas9 with high mutation efficiency derived by expansion of single cells. A DAPI filter was used to detect the signal of Hoechst-stained DNA. Scale bar, 100 μm. (D) Two-cell mouse embryos generated by injection of AG-haESCs into oocytes. The donor haESCs carried Cas9 and sgRNA targeting *Fgf10*. Scale bar, 100 μm. (E) Sanger sequencing analysis of one represented SC mouse carrying biallelic mutant *Fgf10* gene, indicated by multiple peaks in the sequence of PCR products. (F) Schematic of the Cas9/sgRNA-targeting sites in *Runx2*. (G) Sanger sequencing of SC pups with *Runx2* gene mutation at *Runx2*-sg1/2/3, respectively. Relative phenotypes of these mice were shown in Fig 1E. (H) Whole-mount staining of SC mice carrying *Runx2* gene mutation at P0. Mice in red box were shown in Fig 1E. AG-haESC, androgenetic haploid embryonic stem cell; Cas9, CRISPR-associated protein 9; DKO-AG-haESC, double knockout androgenetic haploid embryonic stem cell; haESC, haploid embryonic stem cell; PAM, protospacer adjacent motif; P0, postnatal day 0; SC, semi-cloned; sgRNA, single guide RNA.
(TIF)

S2 Fig CRISPR-Cas9–mediated genetic screening in mice with Cas9B-3 carrying an sgRNA library. (A) Expression level of genes in BD library during osteoblast differentiation. The original data from BioGPS are shown in S2 Table. Genes with an expression level more than 2-fold greater than the median were shown in line 1. Genes with relatively specific expression in osteoblast were shown in line 2. Genes with the expression more than 2-fold increase or decrease during osteoblast differentiation relative to osteoblast_day5 were shown in line 3. (B) Sanger sequencing of the SC mice with homozygous or biallelic mutation generated from Cas9B-3-BD cells in the first 3 ICAHCI experiments. (C) Whole-mount staining of SC mice generated from Cas9B-3-BD by ICAHCI. Mice with abnormalities (score ≥ 2) are marked in a red box. Mice carrying constitutively expressed Cas9 and sgRNA backbone (blank) are marked in a yellow box. BD, bone development related; BioGPS, gene portal system; Cas9, CRISPR-associated protein 9; CRISPR, clustered regularly interspaced palindromic repeats; ICAHCI, intracytoplasmic AG-haESCs injection; SC, semi-cloned; sgRNA, single guide RNA.
(TIF)

S3 Fig Genotype and phenotype analysis of Rln1 and Irx5 knockout mice generated by zygote injection. (A) Genotyping of F1 mice with *Rln1* homozygous mutation. Deletions are indicated with (−). (B) Genotyping of 2 *Irx5* knockout mouse lines with HM 1bp Ins or HM 7bp Del in *Irx5* gene. Deletions are indicated with (−). Insertions are labeled in red. (C–G) μCT analysis of distal femoral metaphysis from 4-week-old mice carrying WT, HM 1bp Ins, or HM 7bp Del in *Irx5* gene. Representative 3D reconstruction of μCT images (C) and for BV/TV (D), Tb.N (E), Tb.Th (F), and C.Th (G). ***P < 0.001, **P < 0.01, *P < 0.05 versus control. Data associated with this figure can be found in S1 Data. BV/TV, bone volume per tissue volume; C.Th, cortical thickness; HM 1bp Ins, homozygous 1-bp insertion; HM 7bp Del, homozygous 7-bp deletion; Tb.N, trabecular number; Tb.Th, trabecular thickness; WT, wild type; μCT, microcomputed tomography.
(TIF)

S4 Fig Irx5 is required for osteogenesis and adipogenesis in vivo. (A) Western blotting analysis of the bone tissue dissected from *Irx5* KO mice. (B) von Kossa staining of vertebra from 4-week-old wild-type and *Irx5* KO mice. Scale bar, 300 μm. (C) Calcein-alizarin red double labeling of vertebra from 4-week-old wild-type and *Irx5* KO mice was visualized by fluorescent microscopy. Scale bar, 100 μm. (D) Immunohistochemistry of OCN of tibiae from 4-week-old wild-type and *Irx5* KO mice. Scale bar, 300 μm (up) and 100 μm (below). (E) TRAP staining of tibiae from 4-week-old wild-type and *Irx5* KO mice. Scale bar, 300 μm. (F) TRAP staining of skulls from 4-week-old wild-type and *Irx5* KO mice. (G–H) TRAP staining of osteoclasts after 6-day culture (G) and culture supernatants were assayed for TRAP activity via colorimetric readout (A405) (H) of bone marrow cells from wild-type and *Irx5* KO mice after 3-day, 6-day, and 7-day culture in the presence of M-CSF and RANKL. Scale bar, 20 μm. (I–J) TRAP staining (I) and TRAP activity (J) of the culture supernatants of wild-type osteoclasts cocultured with wild-type and *Irx5* KO osteoblast progenitors. Scale bar, 20 μm. (K–L) HE staining and Oil Red staining of WAT (K) and BAT (L) from 20-week-old wild-type and *Irx5* KO mice. Scale bar, 100 μm. (M) Mass of epididymal WAT and BAT relative to bodyweight. Data associated with this figure can be found in S1 Data. BAT, brown adipocyte tissue; HE, hematoxylin–eosin; KO, knockout; M-CSF, macrophage colony-stimulating factor; OCN, osteocalcin; RANKL, receptor activator of nuclear factor kappa-B ligand; TRAP, tartrate-resistant acid phosphatase; WAT, white adipocyte tissue.
(TIF)

S5 Fig Irx5 promotes osteogenesis and inhibits adipogenesis in vitro. (A) Gene expression levels of *Irx5*, *Alp*, and *Osterix* in wild-type BMSCs after 0-day, 4-day, 7-day, and 10-day culture in osteoblast differentiation medium examined by qPCR. (B) Gene expression levels of *Irx5*, *Cebpa*, and *Pparg* in wild-type BMSCs after 0-day, 1-day, 3-day, and 5-day culture in adipocyte differentiation medium examined by qPCR. (C) Gene expression of osteoblast marker genes confirmed by qPCR in the differentiated osteoblast cells from wild-type and *Irx5* knockout BMSCs. (D) Gene expression of adipocyte marker genes confirmed by qPCR in the differentiated osteoblast cells from wild-type and *Irx5* knockout BMSCs. (E) Gene expression of *Pparg* target genes by qPCR in long bones from wild-type and *Irx5* knockout mice. Data associated with this figure can be found in S1 Data. BMSC, bone marrow mesenchymal stem cell; qPCR, quantitative polymerase chain reaction.
(TIF)

S6 Fig Irx5 promotes osteogenesis and inhibits adipogenesis by suppressing PPARγ activation. (A) Expression levels of *Pparg* examined by qPCR in wild-type and *Irx5* knockout BMSCs infected with lentivirus expressing EGFP control or PPARγ shRNAs. shEGFP-2, shPPARγ-3, and shPPARγ-4 were selected for further analysis. (B) ALP staining of osteoblasts cultured for 7 days in the wild-type and *Irx5* knockout BMSCs infected with lentivirus expressing EGFP control or PPARγ shRNAs. Scale bar, 50 μm. (C) Statistical analysis of ALP activity (A405) and Alamar Blue activity of the osteoblasts cultured for 7 days. Data are presented as mean ± SD, *n* = 3 in each group. (D) Gene expression levels of *Pparg*, *Runx2*, and *Alp* in osteoblast cultures were examined by qPCR. Data are presented as mean ± SD, *n* = 3 in each group. (E) Oil Red staining of adipocytes cultured for 6 days in the wild-type and *Irx5* knockout BMSCs infected with lentivirus expressing EGFP control or PPARγ shRNAs. Scale bar, 10 μm. (F) Statistical analysis of percentage of Oil Red positive area via Image J. Data are presented as mean ± SD, *n* = 4 in each group. (G) Gene expression levels of *Pparg*, *Perilipin*, and *Lpl* in adipocyte cultures were examined by qPCR. Data are presented as mean ± SD, *n* = 3 in each group. **P < 0.01; *P < 0.05 versus control. Data associated with this figure can be found in S1 Data. ALP, alkaline phosphatase; BMSC, bone marrow mesenchymal stem cell; EGFP, enhanced green fluorescent protein; PPARγ, peroxisome proliferator activated receptor γ; qPCR, quantitative polymerase chain reaction; shEGFP, EGFP shRNA; shPPARγ, PPARγ shRNA; shRNA, short hairpin RNA.
(TIF)

S1 Table Genotype and phenotype analysis of SC mice generated from DKO-AG-haESCs carrying constitutively expressed Cas9 and sgRNA targeting Runx2. SC mice carrying targeted gene homozygous or biallelic mutation with >2 types of alleles detected are labeled in blue; SC mice carrying targeted gene homozygous or biallelic mutation with ≤2 types of alleles detected are labeled in red; SC mice carrying targeted gene homozygous or biallelic mutation without in-frame indels detected are in bold. Cas9, CRISPR-associated protein 9; DKO-AG-haESC, double knockout androgenetic haploid embryonic stem cell; SC, semi-cloned; sgRNA, single guide RNA.
(XLSX)

S2 Table Information on gene expression and protein subcellular localization of 72 genes in BD Library. BD, bone development related.
(XLSX)

S3 Table Information of sgRNAs in BD Library. BD, bone development related; sgRNA, single guide RNA.
(XLSX)

S4 Table Distribution of sgRNAs in Cas9B-3-BD library based on deep sequencing. BD, bone development related; Cas9, CRISPR-associated protein 9; sgRNA, single guide RNA.
(XLSX)

S5 Table Analysis of cell clones randomly collected from Cas9B-3-BD. SgRNAs targeted by 1 time: 26 (in orange shade); sgRNAs targeted by 2 times: 4 (in blue shade); sgRNAs totally targeted: 30; genes totally targeted: 29. BD, bone development related; Cas9, CRISPR-associated protein 9; sgRNA, single guide RNA.
(PDF)

S6 Table Probability of a number of SC mice generated to cover a number of genes in BD library using multinomial distribution analysis. BD, bone development related; SC, semi-cloned.
(PDF)

S7 Table Summary of 426 SC pups generated from Cas9B-3-BD library. Genes targeted by 1 sgRNA: 28 (in orange shade); genes targeted by 2 different sgRNAs: 33 (in blue shade); genes targeted by 3 different sgRNAs: 11 (in green shade); genes totally targeted: 72. BD, bone development related; Cas9, CRISPR-associated protein 9; SC, semi-cloned; sgRNA, single guide RNA.
(XLSX)

S8 Table μCT analysis of Irx5 knockout mice. μCT, microcomputed tomography.
(PDF)

S9 Table Off-target analysis of the sgRNA targeting Irx5. PAM is marked in red; mismatch is marked in orange. PAM, protospacer adjacent motif; sgRNA, single guide RNA.
(PDF)

S10 Table Comparison of approaches for CRISPR/Cas9-mediated genetic screening in mice. CRISPR/Cas9, clustered regularly interspaced palindromic repeats/CRISPR-associated protein 9.
(XLSX)

S11 Table Comparison of mutation efficiency in different approaches of CRISPR/Cas9-mediated genome editing in mouse embryos. a, Embryos were analyzed at E3.5 without transplantation; CRISPR/Cas9, clustered regularly interspaced palindromic repeats/CRISPR-associated protein 9; E, embryonic day; ND, not determined.
(XLSX)

S12 Table Summary of primers. (XLSX)

S1 Data Numerical data used in Figs 1, 2, 3, 4 and 5; S3, S4, S5 and S6 Figs. (XLSX)

## Abbreviations

AG-haESC: androgenetic haploid embryonic stem cell
ALP: alkaline phosphatase
BD: bone development related
BFR: bone formation rate
BioGPS: gene portal system
BMM: bone marrow-derived macrophage
BMSC: bone marrow mesenchymal stem cell
bp: base pair
BV/TV: bone volume per tissue volume
C/EBPβ: CCAAT/enhancer binding protein beta
C/EBPδ: CCAAT/enhancer binding protein delta
cGMP-PKG: cGMP-dependent protein kinase
CLEC11A: c-type lectin domain family 11
CRISPR-Cas9: clustered regularly interspaced palindromic repeats/CRISPR-associated protein 9
C.Th: cortical thickness
C3H10T1/2: murine mesenchymal stem cell line
DAB: diaminobenzidine
DAPI: 4ʹ,6-diamidino-2-phenylindole
DKO: double knockout
dpc: days post coitum; ECM, extracellular matrix
EGFP: enhanced green fluorescent protein
ENU: N-ethyl-N-nitrosourea
FACS: fluorescence-activated cell sorting
FBS: fetal bovine serum
GAPDH: glyceraldehyde-3-phosphate dehydrogenase
GeCKO: genome-scale CRISPR knockout
GO: gene ontology
GSEA: gene set enrichment analysis
HA: hemagglutinin
haESC: haploid embryonic stem cell
HIF-1: hypoxia-inducible factor 1
HPRT: hypoxanthine guanine phosphoribosyl transferase
HE: hematoxylin–eosin
IB: immunoblotting
IBMX: 3-isobutyl-1-methylxanthine
ICAHCI: intracytoplasmic AG-haESCs injection
IP: Immunoprecipitation
IRX5: iroquois homeobox 5
Jak-STAT: Janus kinase/signal transducers and activators of transcription
KEGG: Kyoto Encyclopedia of Genes and Genomes
MAPK: mitogen-activated protein kinases
MAR: mineral apposition rate
M-CSF: macrophage colony-stimulating factor
MOI: multiplicity of infection
M.O.M.: mouse-on-mouse
MSC: mesenchymal stem cell
NF: nuclear factor
OCN: osteocalcin
OPN: osteopontin
PAGE: polyacrylamide gel electrophoresis
PAM: protospacer adjacent motif
PFA: paraformaldehyde
PI3K-AKT: phosphatidylinositol 3-kinase- Protein Kinase B
PPARγ: peroxisome proliferator activated receptor γ
*Pparg-luc*: *Pparg* promoter-luciferase
P0: postnatal day 0
qPCR: quantitative polymerase chain reaction
RANKL: receptor activator of nuclear factor kappa-B ligand
Rap1: Ras-proximate-1
RIG-I: retinoic acid-inducible gene I
RLU: relative light unit
RNAi: RNA interference
RNP: ribonucleoprotein
RNA-seq: RNA sequencing
Runx2: runt related transcription factor 2
SC: semi-cloned
sgRNA: single guide RNA
shRNA: short hairpin RNA
siRNA: small interfering RNA
TA: thymine and adenine
TGF: transforming growth factor
Tb.N: trabecular number
Tb.Th: trabecular thickness
TIDE: tracking of indels by decomposition
TNF: tumor necrosis factor
TRAP: tartrate-resistant acid phosphatase
Wnt: Wingless/Integrated
μCT: microcomputed tomography
293FT: human embryonic kidney cell line
