# Supplementary figures and images for: Targeted genetic screening in mice through haploid embryonic stem cells identifies critical genes in bone development
Source: PLoS Biol. 2019 Jul 2;17(7):e3000350. doi: 10.1371/journal.pbio.3000350 (PMC6629148; doi:10.1371/journal.pbio.3000350)

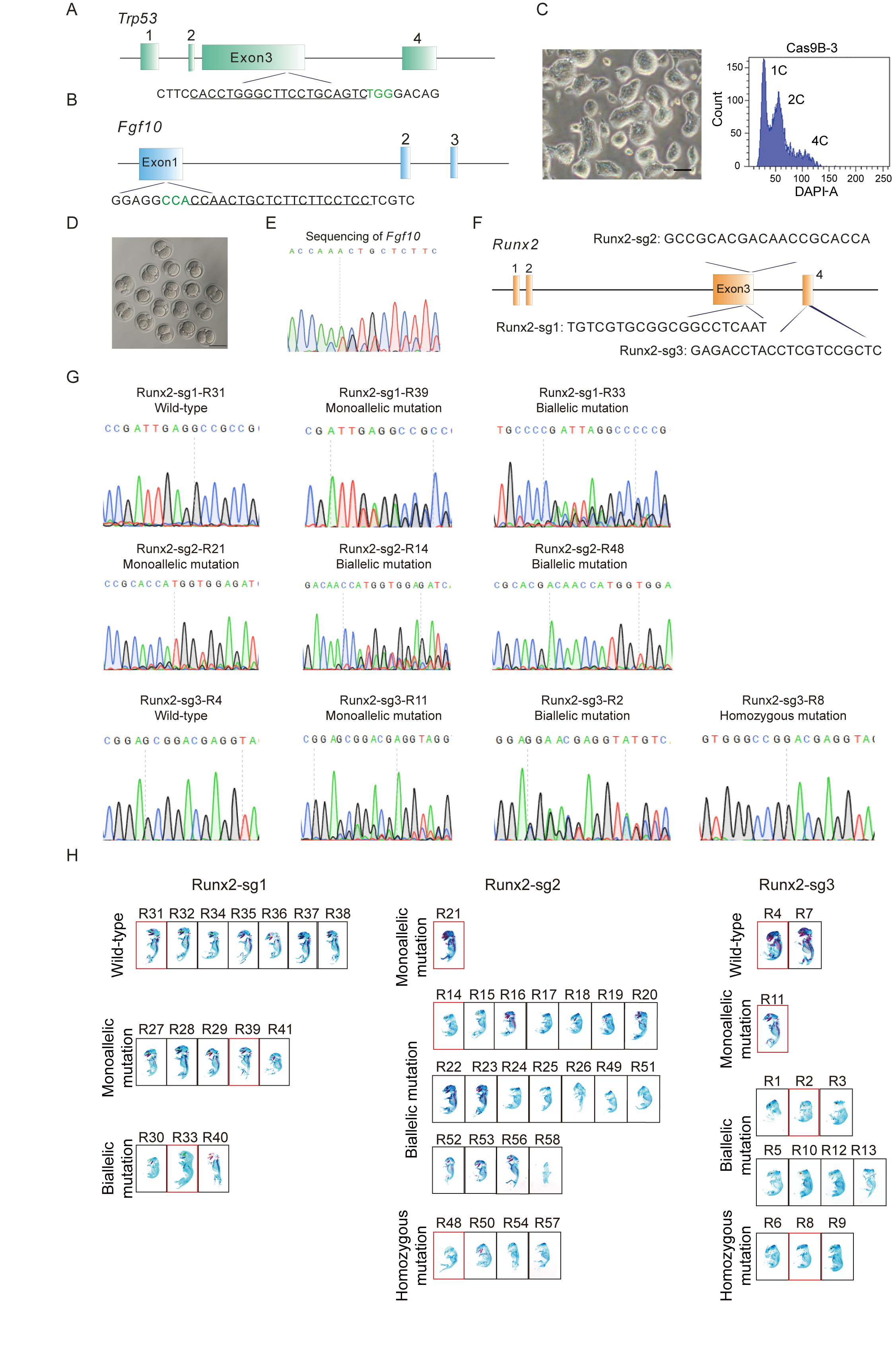

Supplement: S1 Fig — (A–B) Schematic of the Cas9/sgRNA-targeting sites in Trp53 (A) and Fgf10 (B). The sgRNA-targeting sequence is underlined, and the PAM sequence is labeled in green. (C) Phase-contrast image (left) and flow analysis (right) of Cas9B-3, a DKO-AG-haESC line carrying constitutively expressed Cas9 with high mutation efficiency derived by expansion of single cells. A DAPI filter was used to detect the signal of Hoechst-stained DNA. Scale bar, 100 μm. (D) Two-cell mouse embryos generated by injection of AG-haESCs into oocytes. The donor haESCs carried Cas9 and sgRNA targeting Fgf10. Scale bar, 100 μm. (E) Sanger sequencing analysis of one represented SC mouse carrying biallelic mutant Fgf10 gene, indicated by multiple peaks in the sequence of PCR products. (F) Schematic of the Cas9/sgRNA-targeting sites in Runx2. (G) Sanger sequencing of SC pups with Runx2 gene mutation at Runx2-sg1/2/3, respectively. Relative phenotypes of these mice were shown in Fig 1E. (H) Whole-mount staining of SC mice carrying Runx2 gene mutation at P0. Mice in red box were shown in Fig 1E. AG-haESC, androgenetic haploid embryonic stem cell; Cas9, CRISPR-associated protein 9; DKO-AG-haESC, double knockout androgenetic haploid embryonic stem cell; haESC, haploid embryonic stem cell; PAM, protospacer adjacent motif; P0, postnatal day 0; SC, semi-cloned; sgRNA, single guide RNA. (TIF) [file pbio.3000350.s001.tif]

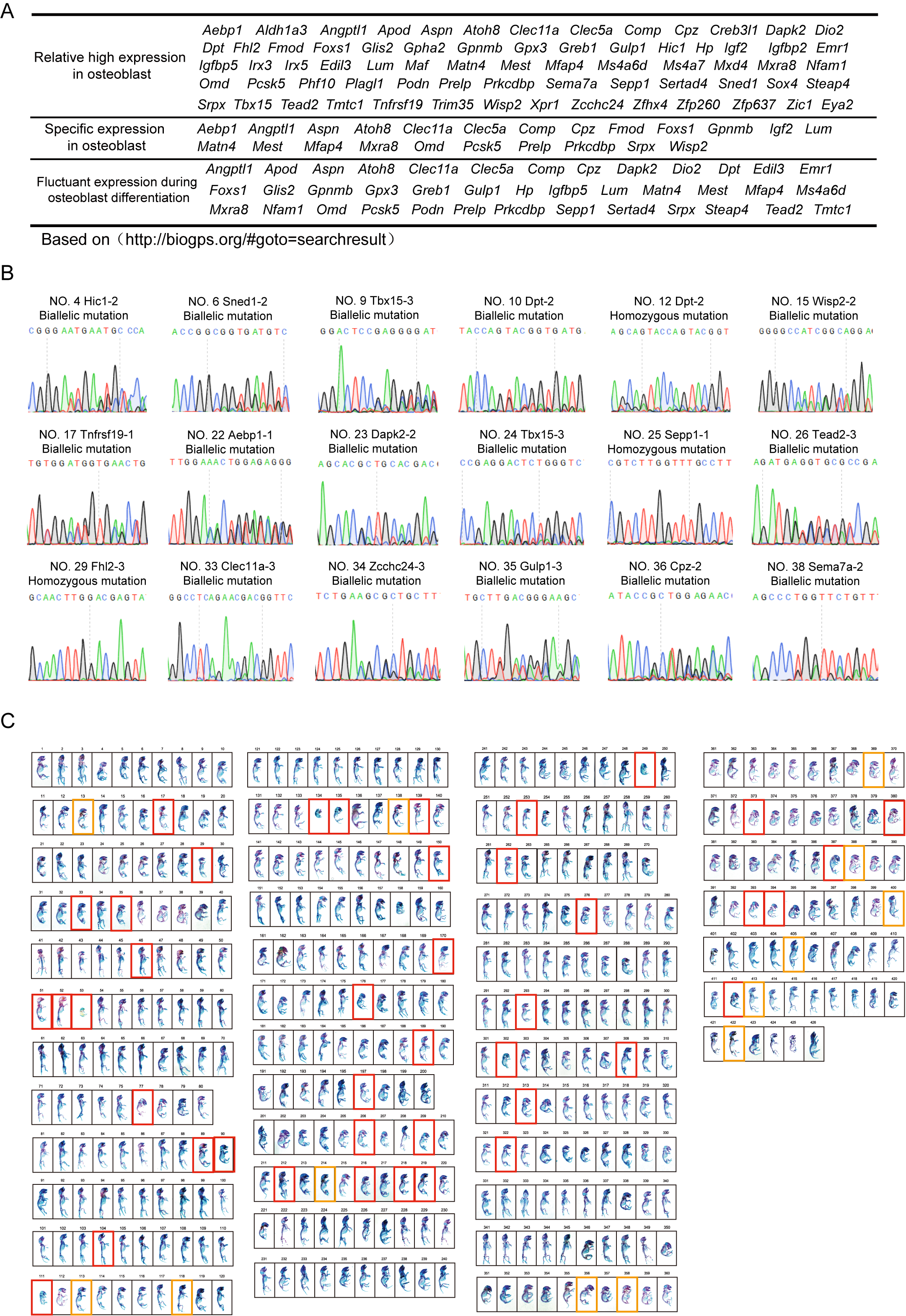

Supplement: S2 Fig — (A) Expression level of genes in BD library during osteoblast differentiation. The original data from BioGPS are shown in S2 Table. Genes with an expression level more than 2-fold greater than the median were shown in line 1. Genes with relatively specific expression in osteoblast were shown in line 2. Genes with the expression more than 2-fold increase or decrease during osteoblast differentiation relative to osteoblast_day5 were shown in line 3. (B) Sanger sequencing of the SC mice with homozygous or biallelic mutation generated from Cas9B-3-BD cells in the first 3 ICAHCI experiments. (C) Whole-mount staining of SC mice generated from Cas9B-3-BD by ICAHCI. Mice with abnormalities (score ≥ 2) are marked in a red box. Mice carrying constitutively expressed Cas9 and sgRNA backbone (blank) are marked in a yellow box. BD, bone development related; BioGPS, gene portal system; Cas9, CRISPR-associated protein 9; CRISPR, clustered regularly interspaced palindromic repeats; ICAHCI, intracytoplasmic AG-haESCs injection; SC, semi-cloned; sgRNA, single guide RNA. (TIF) [file pbio.3000350.s002.tif]

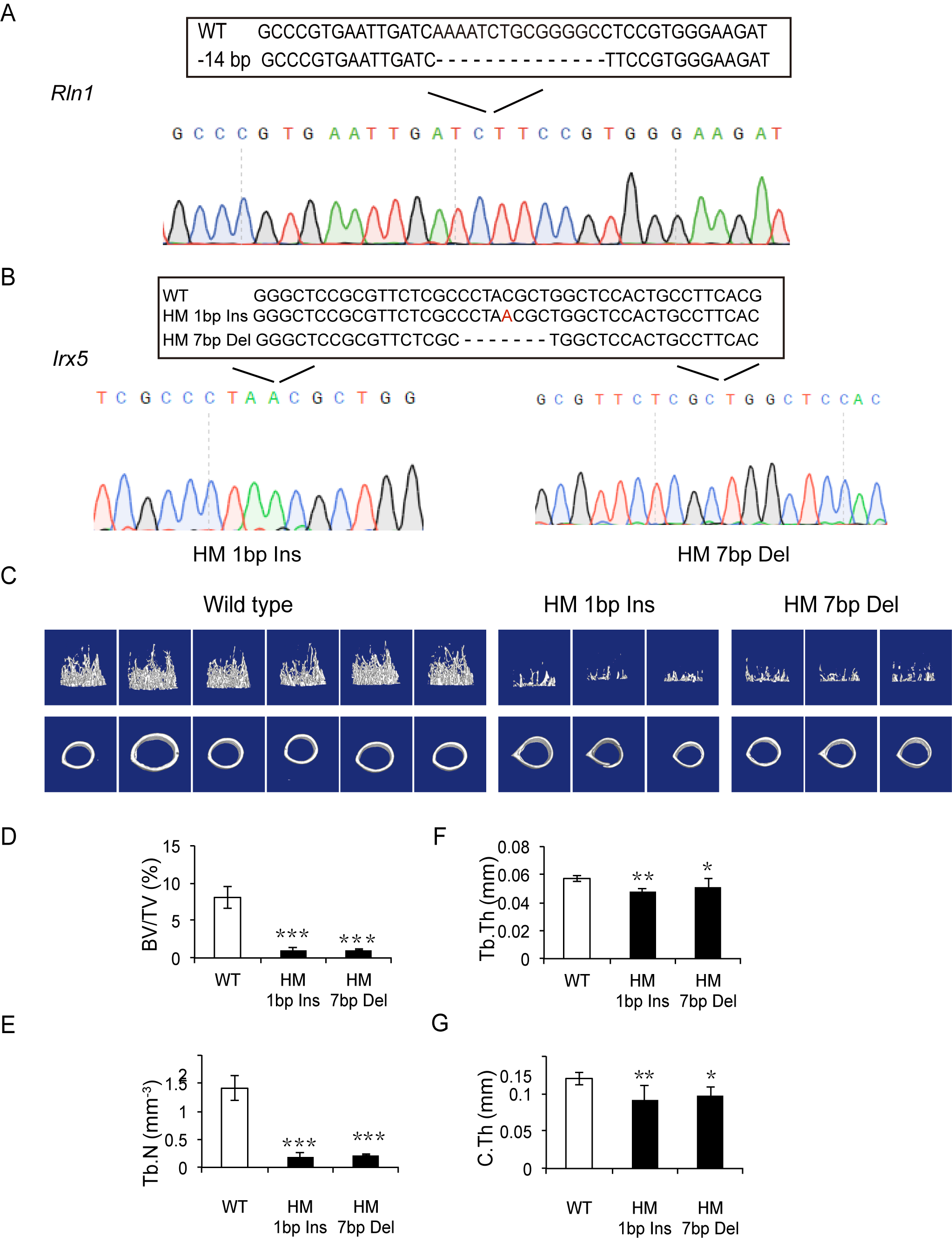

Supplement: S3 Fig — (A) Genotyping of F1 mice with Rln1 homozygous mutation. Deletions are indicated with (−). (B) Genotyping of 2 Irx5 knockout mouse lines with HM 1bp Ins or HM 7bp Del in Irx5 gene. Deletions are indicated with (−). Insertions are labeled in red. (C–G) μCT analysis of distal femoral metaphysis from 4-week-old mice carrying WT, HM 1bp Ins, or HM 7bp Del in Irx5 gene. Representative 3D reconstruction of μCT images (C) and for BV/TV (D), Tb.N (E), Tb.Th (F), and C.Th (G). ***P < 0.001, **P < 0.01, *P < 0.05 versus control. Data associated with this figure can be found in S1 Data. BV/TV, bone volume per tissue volume; C.Th, cortical thickness; HM 1bp Ins, homozygous 1-bp insertion; HM 7bp Del, homozygous 7-bp deletion; Tb.N, trabecular number; Tb.Th, trabecular thickness; WT, wild type; μCT, microcomputed tomography. (TIF) [file pbio.3000350.s003.tif]

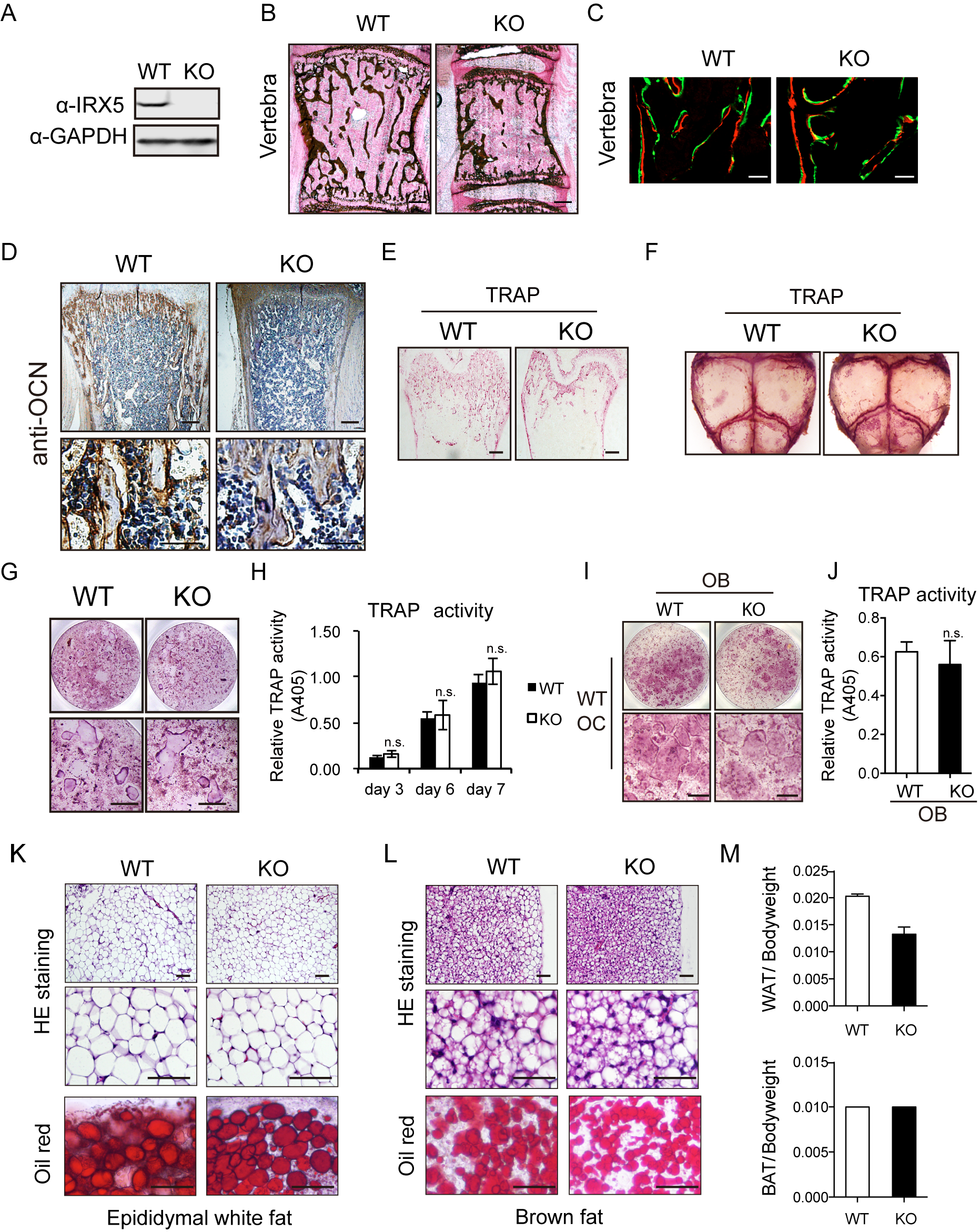

Supplement: S4 Fig — (A) Western blotting analysis of the bone tissue dissected from Irx5 KO mice. (B) von Kossa staining of vertebra from 4-week-old wild-type and Irx5 KO mice. Scale bar, 300 μm. (C) Calcein-alizarin red double labeling of vertebra from 4-week-old wild-type and Irx5 KO mice was visualized by fluorescent microscopy. Scale bar, 100 μm. (D) Immunohistochemistry of OCN of tibiae from 4-week-old wild-type and Irx5 KO mice. Scale bar, 300 μm (up) and 100 μm (below). (E) TRAP staining of tibiae from 4-week-old wild-type and Irx5 KO mice. Scale bar, 300 μm. (F) TRAP staining of skulls from 4-week-old wild-type and Irx5 KO mice. (G–H) TRAP staining of osteoclasts after 6-day culture (G) and culture supernatants were assayed for TRAP activity via colorimetric readout (A405) (H) of bone marrow cells from wild-type and Irx5 KO mice after 3-day, 6-day, and 7-day culture in the presence of M-CSF and RANKL. Scale bar, 20 μm. (I–J) TRAP staining (I) and TRAP activity (J) of the culture supernatants of wild-type osteoclasts cocultured with wild-type and Irx5 KO osteoblast progenitors. Scale bar, 20 μm. (K–L) HE staining and Oil Red staining of WAT (K) and BAT (L) from 20-week-old wild-type and Irx5 KO mice. Scale bar, 100 μm. (M) Mass of epididymal WAT and BAT relative to bodyweight. Data associated with this figure can be found in S1 Data. BAT, brown adipocyte tissue; HE, hematoxylin–eosin; KO, knockout; M-CSF, macrophage colony-stimulating factor; OCN, osteocalcin; RANKL, receptor activator of nuclear factor kappa-B ligand; TRAP, tartrate-resistant acid phosphatase; WAT, white adipocyte tissue. (TIF) [file pbio.3000350.s004.tif]

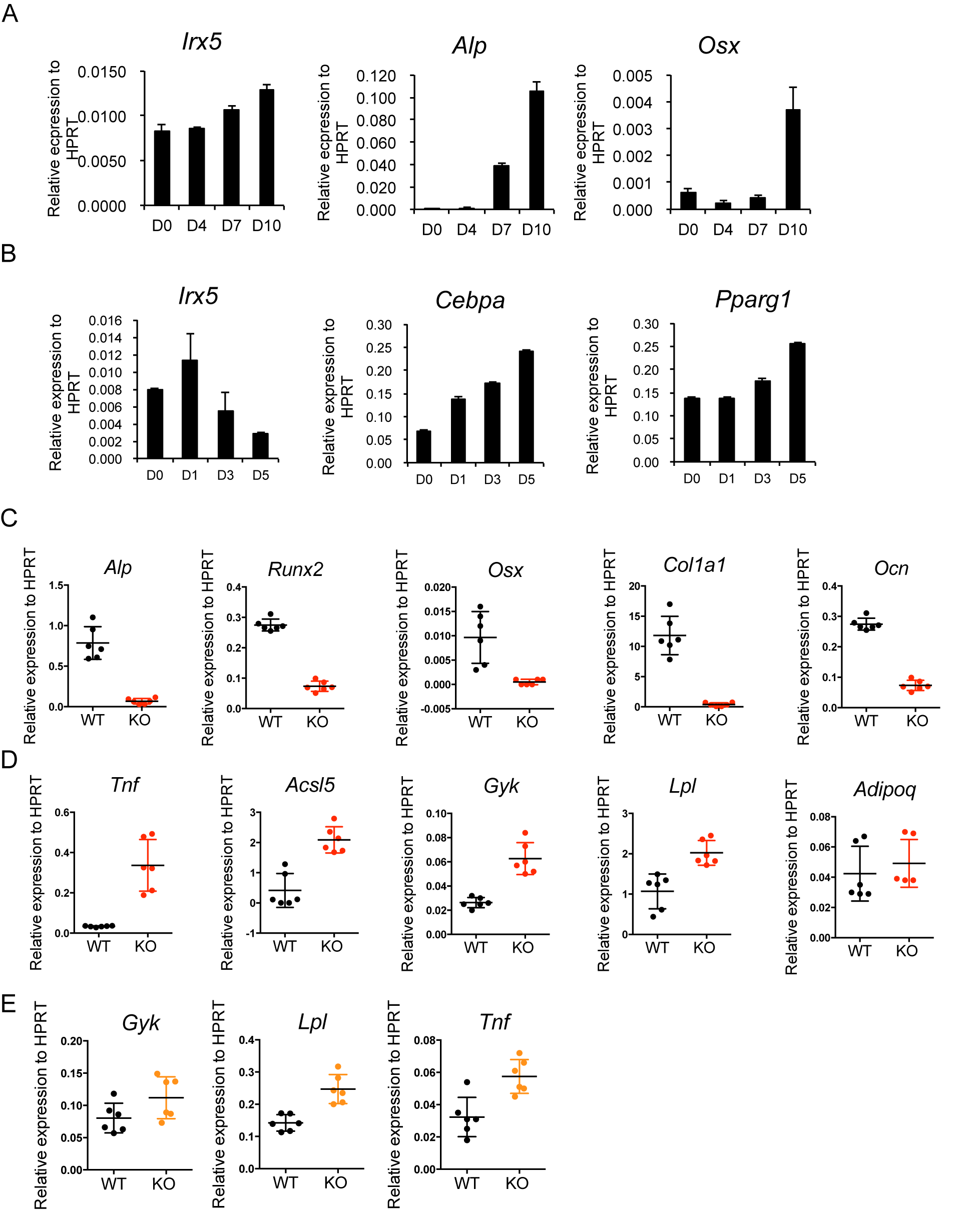

Supplement: S5 Fig — (A) Gene expression levels of Irx5, Alp, and Osterix in wild-type BMSCs after 0-day, 4-day, 7-day, and 10-day culture in osteoblast differentiation medium examined by qPCR. (B) Gene expression levels of Irx5, Cebpa, and Pparg in wild-type BMSCs after 0-day, 1-day, 3-day, and 5-day culture in adipocyte differentiation medium examined by qPCR. (C) Gene expression of osteoblast marker genes confirmed by qPCR in the differentiated osteoblast cells from wild-type and Irx5 knockout BMSCs. (D) Gene expression of adipocyte marker genes confirmed by qPCR in the differentiated osteoblast cells from wild-type and Irx5 knockout BMSCs. (E) Gene expression of Pparg target genes by qPCR in long bones from wild-type and Irx5 knockout mice. Data associated with this figure can be found in S1 Data. BMSC, bone marrow mesenchymal stem cell; qPCR, quantitative polymerase chain reaction. (TIF) [file pbio.3000350.s005.tif]

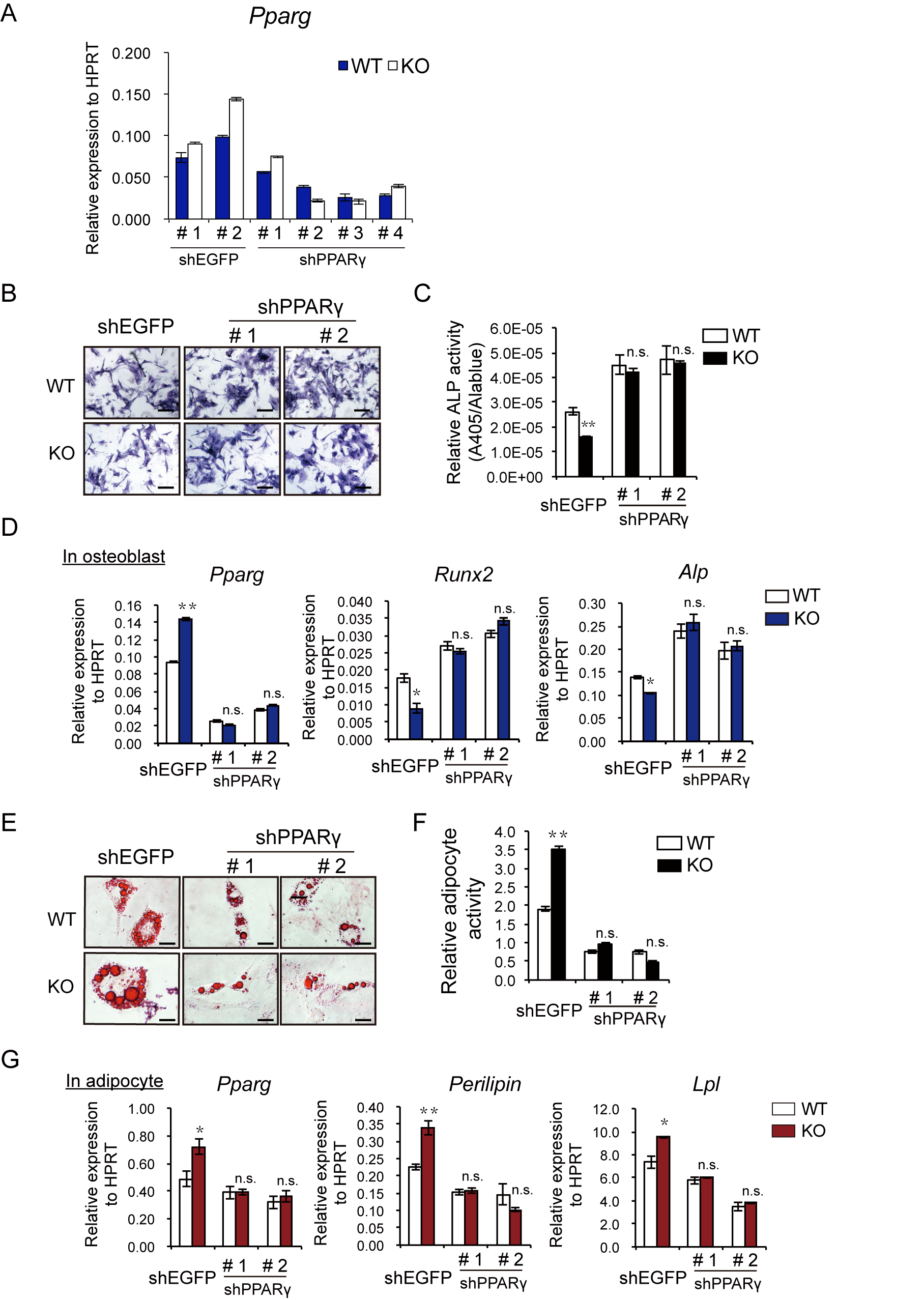

Supplement: S6 Fig — (A) Expression levels of Pparg examined by qPCR in wild-type and Irx5 knockout BMSCs infected with lentivirus expressing EGFP control or PPARγ shRNAs. shEGFP-2, shPPARγ-3, and shPPARγ-4 were selected for further analysis. (B) ALP staining of osteoblasts cultured for 7 days in the wild-type and Irx5 knockout BMSCs infected with lentivirus expressing EGFP control or PPARγ shRNAs. Scale bar, 50 μm. (C) Statistical analysis of ALP activity (A405) and Alamar Blue activity of the osteoblasts cultured for 7 days. Data are presented as mean ± SD, n = 3 in each group. (D) Gene expression levels of Pparg, Runx2, and Alp in osteoblast cultures were examined by qPCR. Data are presented as mean ± SD, n = 3 in each group. (E) Oil Red staining of adipocytes cultured for 6 days in the wild-type and Irx5 knockout BMSCs infected with lentivirus expressing EGFP control or PPARγ shRNAs. Scale bar, 10 μm. (F) Statistical analysis of percentage of Oil Red positive area via Image J. Data are presented as mean ± SD, n = 4 in each group. (G) Gene expression levels of Pparg, Perilipin, and Lpl in adipocyte cultures were examined by qPCR. Data are presented as mean ± SD, n = 3 in each group. **P < 0.01; *P < 0.05 versus control. Data associated with this figure can be found in S1 Data. ALP, alkaline phosphatase; BMSC, bone marrow mesenchymal stem cell; EGFP, enhanced green fluorescent protein; PPARγ, peroxisome proliferator activated receptor γ; qPCR, quantitative polymerase chain reaction; shEGFP, EGFP shRNA; shPPARγ, PPARγ shRNA; shRNA, short hairpin RNA. (TIF) [file pbio.3000350.s006.tif]
