# Supplementary material for: Targeted genetic screening in mice through haploid embryonic stem cells identifies critical genes in bone development
Source: PLoS Biol. 2019 Jul 2;17(7):e3000350. doi: 10.1371/journal.pbio.3000350 (PMC6629148; doi:10.1371/journal.pbio.3000350)
Supplement: S5 Table — SgRNAs targeted by 1 time: 26 (in orange shade); sgRNAs targeted by 2 times: 4 (in blue shade); sgRNAs totally targeted: 30; genes totally targeted: 29. BD, bone development related; Cas9, CRISPR-associated protein 9; sgRNA, single guide RNA. (PDF) [file pbio.3000350.s011.pdf]

S5 Table Analysis of cell clones randomly collected from Cas9B-3-BD.

Clone NO.	Gene-sgRNA	sgRNA-Sequence	Mutation Status
1	*Clec11a*-1	TCAGCCCAATGGCGGCGTCC	Wild-type
2	*Edil3*-3	TCCACAACACTAGAGCAGTT	Mutation
3	*Dapk2*-2	CTCAAGGATGAGCACCACGT	Mutation
4	*Dpt*-2	ATCCATCATCACCGTAGTCC	Mutation
5	*Fhl2*-3	TGAATACTCGTCCAAGTGCC	Mutation
6	*Glis2*-1	AAGCGTCCGTCCACCTTCTC	Wild-type
7	*Gulp1*-3	AAGTTGTGAGAGATGCTGTC	Wild-type
8	*Fmod*-2	AGGGTCATAGGGGTCGTAGT	Mutation
9	*Irx3*-2	CCTCCTCGTCCGTGCGACTA	Mutation
10	*Lum*-1	GTGGACCGACGGACTCGGTC	Mutation
11	*Maf*-1	GCACTTCGACGACCGCTTCT	Mutation
12	*Greb1*-1	CAGCAGCTGTCATCGTTAGA	Mutation
13	*Ms4a7*-2	GGTACTTACCCCAGGTTTGT	Mutation
14	*Sema7a*-2	CAGCCCGGATGAGAACTCCC	Mutation
15	*Omd*-3	CCATTTAACATACCTTCGTG	Mutation
16	*Plagl1*-2	GCTCCCGAGAACGGGCTTGA	Mutation
17	*Podn*-2	CGGGGTAGATCTTCTCCAGC	Wild-type
18	*Sepp1*-1	GGCCGTCTTGTGTATCACCT	Mutation
19	*Zbtb32*-1	TCGGCCTCGAGGAAGAGTAG	Mutation
20	*Srpx*-3	GACTCACCACTAGAAGACGA	Mutation
21	*Steap4*-2	CTATCCGTTACTATGTTCGA	Mutation
22	*Tbx15*-3	GGTCCGCTCGGTGAGCACCT	Mutation
23	*Tead2*-3	GACGATGGCCACCATGTCTT	Wild-type
24	*Tnfrsf19*-1	ACCAGCGCACAGTCCGCACA	Mutation
25	*Gulp1*-3	AAGTTGTGAGAGATGCTGTC	Mutation
26	*Srpx*-3	GACTCACCACTAGAAGACGA	Wild-type
27	Blank	GGGTCTTCGAGAAGAC	-
28	*Fhl2*-3	TGAATACTCGTCCAAGTGCC	Mutation
29	*Dio2*-3	ACCTACAAGAAGTCCGAAGT	Mutation
30	*Aebp1*-1	TAACCGATCCAGCGTTTCCA	Mutation
31	*Tmtc1*-3	CCATGTCGCCGAGAGCTATG	Mutation
32	*Clec11a*-3	CCACCAAGAACCGTCGTCTG	Mutation
33	*Foxs1*-3	CCTCGTGATGACCGCAAGCC	Mutation
34	*Edil3*-3	TCCACAACACTAGAGCAGTT	Wild-type
35	*Trim35*-2	TGGTGTAGGTGCAAACGACT	Mutation
	Mutation Efficiency		77.1% (27/35)

SgRNAs targeted by 1 time: 26 (yellow)
SgRNAs targeted by 2 times: 4 (blue)
SgRNAs totally targeted: 30
Genes totally targeted: 29
