# Supplementary material for: Targeted genetic screening in mice through haploid embryonic stem cells identifies critical genes in bone development
Source: PLoS Biol. 2019 Jul 2;17(7):e3000350. doi: 10.1371/journal.pbio.3000350 (PMC6629148; doi:10.1371/journal.pbio.3000350)
Supplement: S6 Table — BD, bone development related; SC, semi-cloned. (PDF) [file pbio.3000350.s012.pdf]

S6 Table Probability of a number of SC mice generated to cover a number of genes in BD Library using multinomial distribution analysis.

Probability		NO. of Mice Needed					
		200	300	400	500	600	700
NO. of Genes Covered	72	0.0%	1.0%	11.5%	33.3%	55.4%	71.7%
	71	0.0%	6.3%	38.4%	71.7%	89.2%	96.0%
	70	0.2%	19.3%	67.2%	91.8%	98.3%	99.7%
	69	0.7%	39.5%	86.9%	98.3%	99.8%	100.0%
	68	2.5%	61.6%	95.9%	99.7%	99.8%	100.0%
	67	7.2%	79.2%	98.9%	100.0%	100.0%	100.0%
	66	15.3%	90.6%	99.8%	100.0%	100.0%	100.0%
	65	28.0%	96.4%	100.0%	100.0%	100.0%	100.0%
	64	44.0%	98.9%	100.0%	100.0%	100.0%	100.0%
	63	60.3%	99.7%	100.0%	100.0%	100.0%	100.0%
	62	74.5%	99.9%	100.0%	100.0%	100.0%	100.0%
	61	85.6%	100.0%	100.0%	100.0%	100.0%	100.0%
	60	92.7%	100.0%	100.0%	100.0%	100.0%	100.0%
	59	96.7%	100.0%	100.0%	100.0%	100.0%	100.0%
	58	98.7%	100.0%	100.0%	100.0%	100.0%	100.0%
	57	99.6%	100.0%	100.0%	100.0%	100.0%	100.0%
	56	99.9%	100.0%	100.0%	100.0%	100.0%	100.0%
	55	100.0%	100.0%	100.0%	100.0%	100.0%	100.0%
	54	100.0%	100.0%	100.0%	100.0%	100.0%	100.0%
	53	100.0%	100.0%	100.0%	100.0%	100.0%	100.0%
