# Supplementary material for: Targeted genetic screening in mice through haploid embryonic stem cells identifies critical genes in bone development
Source: PLoS Biol. 2019 Jul 2;17(7):e3000350. doi: 10.1371/journal.pbio.3000350 (PMC6629148; doi:10.1371/journal.pbio.3000350)
Supplement: S8 Table — μCT, microcomputed tomography. (PDF) [file pbio.3000350.s014.pdf]

S8 Table μ-CT analysis of *Irx5* knockout mice.

Genotype		Gender	weight/g	Percent bone volume BV/TV	Trabecular thickness Tb.Th	Trabecular number Tb.N	Cortical thikness C.Th
Irx5 WT		M	12.9	10.41	0.06	1.73	0.13
Irx5 WT		M	13.6	12.72	0.06	2.02	0.12
Irx5 WT		M	10.9	9.71	0.06	1.65	0.13
Irx5 WT		M	15.2	7.30	0.05	1.35	0.12
Irx5 WT		M	10.6	8.93	0.06	1.51	0.11
Irx5 WT		M	10.9	6.36	0.06	1.13	0.12
Irx5 HM	1bp Insertion	M	6	1.03	0.05	0.21	0.08
Irx5 HM	1bp Insertion	M	5.5	0.54	0.05	0.12	0.08
Irx5 HM	1bp Insertion	M	6.5	1.30	0.05	0.26	0.11
Irx5 HM	7bp Deletion	M	6.1	1.25	0.06	0.21	0.11
Irx5 HM	7bp Deletion	M	5.1	1.09	0.05	0.23	0.10
Irx5 HM	7bp Deletion	M	6.3	0.95	0.05	0.21	0.08
