# Supplementary material for: Targeted genetic screening in mice through haploid embryonic stem cells identifies critical genes in bone development
Source: PLoS Biol. 2019 Jul 2;17(7):e3000350. doi: 10.1371/journal.pbio.3000350 (PMC6629148; doi:10.1371/journal.pbio.3000350)
Supplement: S9 Table — PAM is marked in red; mismatch is marked in orange. PAM, protospacer adjacent motif; sgRNA, single guide RNA. (PDF) [file pbio.3000350.s015.pdf]

S9 Table Off-target analysis of the sgRNA targeting *Irx5*.

Locus	Sequence	Genomic Coordinates	% Indel (mutant/total)
OT-1	CCCGGGTTCTCACCCTACGTTAG	GRCm38/mm10_chr13:41188069-41188091	0 (0/12)
OT-2	TCCGTGTCCTCTCCCTTCGCAGG	GRCm38/mm10_chr5:66802250-66802272	0 (0/12)
OT-3	TCCAAGTTCTTGCCCTACTCAAG	GRCm38/mm10_chr15:53947738-53947760	0 (0/12)
OT-4	CCTGCGTTCTCTCCCTACCCTGG	GRCm38/mm10_chr4:142133046-142133068	0 (0/12)
OT-5	GCTGCGTTCTCGCCCTGCTCCAG	GRCm38/mm10_chr17:56141816-56141838	0 (0/12)
OT-6	TCTGCGTTCCCGCCCTACAGCAG	GRCm38/mm10_chr10:80434082-80434104	0 (0/12)
OT-7	TCCGCCTTCTCCCCCTCCGTGGG	GRCm38/mm10_chr2:163645181-163645203	0 (0/12)
OT-8	TCCGTGTTCTGGCCCTCAGCGGG	GRCm38/mm10_chr3:116595403-116595425	0 (0/12)
OT-9	AGCGCCTTCTTGCCCTATGCCGC	GRCm38/mm10_chr13:71963238-71963260	0 (0/12)
OT-10	GGCGCCTTCCTGCCCTACGCCAC	GRCm38/mm10_chr8:91800990-91801012	0 (0/12)
OT-11	TCCCCTTGCCCACTCTACGATTC	GRCm38/mm10_chr13:73265423-73265445	0 (0/12)
OT-12	TCCTCCTGCTCACCCACCCCTGC	GRCm38/mm10_chr8:92679952-92679974	0 (0/12)

PAM: Marked in red
Mismatch: Marked in orange
